# ﻿Two new species and a new genus of ray spiders (Araneae, Theridiosomatidae) from the Ryukyu Islands, southwest Japan, with notes on their natural history

**DOI:** 10.3897/zookeys.1109.83807

**Published:** 2022-07-01

**Authors:** Yuya Suzuki, Takehisa Hiramatsu, Haruki Tatsuta

**Affiliations:** 1 The United Graduate School of Agricultural Sciences, Kagoshima University, 1-21-24, Korimoto, Kagoshima-shi, Kagoshima, 890-0065, Japan Kagoshima University Kagoshima Japan; 2 Graduate School of Systems Life Sciences, Kyushu University, 744 Motooka, Nishi-ku, Fukuoka, 819-0395 Japan Kyushu University Motooka Japan; 3 Fregrance-Uwado 203, Uwado, Kawagoe-shi, Saitama, 350-0816, Japan Unaffiliated Saitama Japan

**Keywords:** Araneoidea, embolic apophysis, limestone cave, Iriomote Island, new combination, Okinawa Island, orb web, taxonomy

## Abstract

This paper provides descriptions of two new theridiosomatid species, *Theridiosomanigrivirgatum***sp. nov.** and *Sennintanikawai***gen. nov.**, **sp. nov.** from the Ryukyu Islands, southwest Japan, with photographs and illustrations of both sexes. *Sennin***gen. nov.** is a troglophilic genus composed of two species, *S.tanikawai***sp. nov.** (Iriomote Island, Japan) and *S.coddingtoni* (Zhu, Zhang & Chen, 2001), **comb. nov.** (southern China). *Zomadibaiyin* Miller, Griswold & Yin, 2009, which recently joined the Japanese fauna, was morphologically reexamined based on specimens from the Ryukyus, and taxonomic features of *Zoma* males were reassessed. A distributional map of theridiosomatid spiders in the Ryukyus is also provided, including *T.dissimulatum* Suzuki, Serita & Hiramatsu, 2020, and *T.alboannulatum* Suzuki, Serita & Hiramatsu, 2020 with their habitat types, web morphology, and web-building behavior in detail.

## ﻿Introduction

The family Theridiosomatidae Simon, 1881 (Araneae: Araneoidea) is composed of small-sized (body length, ca. 0.5–3 mm) spiders that prefer dark and humid environments such as forest floors, mountainous streams, and caves (Coddington 1986a). The family is characterized by a sternal pit organ (Wunderlich 1980) that has a pair of pits on the anterior margin of the sternum, except in *Chthonos* Coddington, 1986 (Coddington 1986a), large globular palp of males, a pair of spermathecae fused or in contact with each other in females except in *Coddingtonia* Miller, Griswold & Yin, 2009 (Miller et al. 2009), and elongated trichobothria on the dorsum of the third and fourth legs (Coddington 1986a). All genera except *Chthonos* are known as web-builders (Coddington 1986a), and several genera build conventional orb webs (e.g., *Baalzebub* Coddington, 1986, *Epeirotypus* O. Pickard-Cambridge, 1894, and *Naatlo* Coddington, 1986), while others construct orb webs with radial anastomosis (e.g., *Theridiosoma* O. Pickard-Cambridge, 1879) or deformed webs, called sparse networks (e.g., *Ogulnius* O. Pickard-Cambridge, 1882, *Wendilgarda* Keyserling, 1886). Webs of some genera are characterized by a tension line, a non-sticky isolated radius that stretches from the center of the web and attaches to the substrates (e.g., *Epeirotypus*, *Naatlo*, and *Theridiosoma*). Spiders of these genera drag the tension line with forelegs and hold it into coiled conditions while holding the web with hindlegs. Therefore, the web acquires a distorted conical shape (Shinkai and Shinkai 1985; Coddington 1986a). When flying insects approach the web, the tension line is promptly released, and the launched web captures the prey. This latch-mediated spring actuation results in an ultrafast web shooting motion (Alexander and Bhamla 2020).

Currently, 19 genera and 133 species of Theridiosomatidae are recorded mainly in tropical and subtropical regions worldwide (World Spider Catalog 2022). After the revision by Coddington (1986a), new genera and new species were described mostly from China and Southeast Asia (Miller et al. 2009; Chen 2010; Wunderlich 2011; Zhao and Li 2012; Labarque and Griswold 2014; Zhao and Li 2014; Prete et al. 2018). Twelve genera, namely *Baalzebub*, *Chthonopes* Wunderlich, 2011, *Coddingtonia*, *Epeirotypus*, *Karstia* Chen, 2010, *Menglunia* Zhao & Li, 2012, *Ogulnius*, *Sinoalaria* Zhao & Li, 2014, *Tagalogonia* Labarque & Griswold, 2014, *Theridiosoma*, *Wendilgarda*, and *Zoma* Saaristo, 1996, were recorded from East to Southeast Asia. These genera are distributed in both neotropics and Asia, with several exceptions such as *Karstia* and *Menglunia*, which are endemic to China, and *Tagalogonia*, endemic to the Philippines (World Spider Catalog 2022).

The Ryukyu Islands, comprising hundreds of continental islands located between Kyushu and Taiwan, were formed by a complicated geological history of several land bridge connections with the Chinese continent. Consequently, the fauna constitutes continental components and consists of many endemic species that have been derived from the continental ancestry (e.g., Ota 1998, 2000). Spider fauna of the Ryukyu Islands has been surveyed by many arachnologists (e.g., Shimojana 1977), but small-sized spiders such as Theridiosomatidae have only recently been examined (e.g., Shinkai and Hiramatsu 2000). Our recent surveys on the islands revealed two new species of *Theridiosoma* from the Ryukyus, and two theridiosomatid species as new members of the spider fauna in the Ryukyus: *Theridiosomadissimulatum* Suzuki, Serita & Hiramatsu, 2020, *T.alboannulatum* Suzuki, Serita & Hiramatsu, 2020, *Wendilgardaruficeps* Suzuki, 2019 and *Zomadibaiyin* Miller, Griswold & Yin, 2009 (Ono and Ogata 2018; Suzuki et al. 2020; Suzuki and Matsushima 2021; Suzuki and Serita 2021).

During our survey in the Ryukyu Islands conducted between 2020 and 2022, several unidentified specimens of theridiosomatid spiders were further discovered from secondary forests, grasslands, and bushes in Okinawa, Kume and Aka Islands, and limestone caves on Iriomote Island. Based on morphological examination, we concluded that these specimens belong to two new species. One species was determined to be an undescribed *Theridiosoma* species. We confirmed that the second undescribed species from caves on Iriomote Island possesses unique characteristics that do not correspond to the taxonomic characteristics of known theridiosomatid genera. We were also aware that *Karstiacoddingtoni* (Zhu, Zhang & Chen, 2001), known from Southern China, shares common features with the undescribed species in Iriomote Island. Here, we suggest the establishment of a new genus named *Sennin* gen. nov., that comprises of these two species. Furthermore, several specimens of the Chinese species *Zomadibaiyin* Miller, Griswold & Yin, 2009, which was recently recognized in the Japanese fauna (Ono and Ogata 2018; Suzuki and Serita 2021), were morphologically reexamined, and taxonomic characteristics of *Zoma* males were reassessed. We provide descriptions of these three theridiosomatid species, including two new species with illustrations and photographs of both sexes. Furthermore, geographical distributional data including other theridiosomatids in the Ryukyus are also provided with a comparison of their natural history, especially habitat types, web morphology, and web-building behavior.

## ﻿Materials and methods

The specimens were preserved in 80% (v/v) ethanol solution. Morphological features of the specimens were observed, and photographs were taken using a stereoscopic microscope (Nikon AZ100M, Japan). Photographed images were stacked using microscope imaging software (Nikon NIS-Elements D 4.20.00 64-bit, Japan). Photographs of *Z.dibaiyin* were taken using a digital camera (Nikon CF Plan X20 objective lens + Olympus M. Zuiko 75–300 mm attached to Olympus OM-D E-M1) and stacked using imaging software (Zerene Stucker; Zerene Systems, Washington, USA). The vulvae were treated with Proteinase K before being photographed. Measurements of the legs are given in the following format: femur + patella + tibia + metatarsus + tarsus = total, in millimeters. The formula of macrosetae on the legs is as follows: **d**, dorsal; **p**, prolateral; **r**, retrolateral. All specimens used in this study were deposited in the collection of the Department of Zoology, National Museum of Nature and Science, Tsukuba (**NSMT**; curator: Ken-ichi Okumura), Tsukuba, Japan. Specimens without registration numbers in the ‘material examined’ section were deposited in the personal collection of YS.

Observations of *Sennintanikawai* sp. nov. were conducted in Yutsun-do cave and Ôtomi-daiichi-do cave on Iriomote Island in August 1998, and April and June 2021. The measurements of webs and visual observations of web-building behavior were conducted using a 6V search light. The web size was measured for the horizontal and vertical diameters of the capture area. The number of sticky spirals was counted along a radius located at an angle of 45° in the upper right sector of the orb. Web-building behavior was observed in the adult females. Webs were photographed and behaviors on the webs were recorded as movie in the spiders’ natural habitat using a digital camera (Laowa 50 mm Ultra Macro + Olympus OM-D-E-M1; Canon DS6041 + Canon Macro Lens EF 100 mm).

Abbreviations of morphological terminology are in accordance with Coddington (1986a), Zhu et al. (2001), Miller et al. (2009), Chen (2010), and Zhao and Li (2012). MAL, MAW, PCP, and RCP are defined herein for the first time; see below for all abbreviations for morphology:

**AL** abdomen length;

**ALE** anterior lateral eye;

**AME** anterior median eye;

**AW** abdomen width;

**C** conductor;

**CA** cymbial apophysis;

**CaL** carapace length;

**CAW** cymbial apophysis width;

**CaW** carapace width;

**CB** copulatory bursae;

**CD** copulatory duct;

**CL** cymbial lamella;

**E** embolus;

**EA** embolic apophysis;

**ED** embolic division;

**ES** epigynal scape;

**ESL** epigynal scape length;

**FD** fertilization duct;

**MA** median apophysis;

**MAL** length of dorsal protrusion on median apophysis;

**MAW** width of dorsal protrusion on median apophysis;

**PC** paracymbium;

**PCP** posterior conductor projection;

**PLE** posterior lateral eye;

**PME** posterior median eye;

**PTL** palpal tibia length;

**RCP** retrolateral conductor projection;

**S** spermatheca;

**ST** subtegulum;

**T** tegulum;

**VW** vulva width.

Abbreviations for web architecture:

**HL** hub loop;

**OH** open hub;

**RA** radial anastomosis;

**RD** radii;

**SS** sticky spirals;

**TL** tension line;

**TS** temporary spiral.

### ﻿Key to the theridiosomatid species in the Ryukyu Islands

**Table:** 

1	Male	**2**
–	Female	**6**
2	Cymbium with a long dorsal cymbial apophysis, embolic apophyses entirely covered with conductor	***Sennintanikawai* sp. nov.**
–	Cymbium lacking cymbial apophysis	**3**
3	One embolic apophysis exposed from conductor	***Zomadibaiyin***
–	Two embolic apophyses exposed from conductor	**4**
4	Conductor with a projection	***Theridiosomadissimulatum***
–	Conductor lacking a projection	**5**
5	Two paralleled embolic apophyses of same length	***Theridiosomaalboannulatum***
–	One embolic apophysis longer than the other	***Theridiosomanigrivirgatum* sp. nov.**
6	Epigyne with a scape on posterior margin	***Sennintanikawai* sp. nov.**
–	Epigyne lacking a scape	**7**
7	Epigyne with a sclerotized median pit	***Zomadibaiyin***
–	Epigyne lacking a sclerotized median pit	**8**
8	Epigyne with an invagination on posterior margin	**9**
–	Epigyne lacking an invagination on posterior margin	***Theridiosomaalboannulatum***
9	Heart-shaped invagination with a pair of spurs	***Theridiosomadissimulatum***
–	Slit-like invagination	***Theridiosomanigrivirgatum* sp. nov.**

## ﻿Taxonomy

### ﻿Family Theridiosomatidae Simon, 1881

#### ﻿Genus Theridiosoma

Taxon classificationAnimaliaAraneaeTheridiosomatidae

O. Pickard-Cambridge, 1879

8B939A91-DEA7-57C6-A494-7D81CBB7F986

##### Type species.

*Theridiosomagemmosum* (L. Koch, 1877), from Nuremberg, West Germany (not examined).

##### Remarks.

Males of *Theridiosoma* species can be distinguished from other theridiosomatid genera by the morphology of the embolic division of the male palp: short and tubular embolus with embolic apophyses fragmented into several long bristle-like parts (Coddington 1986a: figs 131, 133). Embolic apophysis varies in number and shape among species and is regarded as an important taxonomic character (e.g., Zhao and Li 2012; Suzuki et al. 2020). Median apophysis is less sclerotized, curved and attenuates distally (Coddington 1986a: figs 132, 133), which is less useful for distinguishing species. A sclerotized projection (‘conductor projection’) is present on prolateral side of conductor in some species, while absent in others (Coddington 1986a; Suzuki et al. 2020). Distal margin of conductor beneath embolic apophyses (‘posterior margin of embolic division’ in Suzuki et al. 2020) is generally sclerotized and the shape is useful as a taxonomic character (e.g., Suzuki et al. 2020: figs 7E, 8E, 10E, 11E). Tegular surface beneath conductor is generally sclerotized with many folds (referred as ‘ventral side of tegulum beneath posterior edge of embolic division’ in Suzuki et al. 2020), of which shape and surface texture vary among species (Zhao and Li 2012; Suzuki et al. 2020).

Females of the genus can be distinguished from related genera (*Baalzebub*, *Epilineutes* and *Wendilgarda*) by having relatively sclerotized, robust copulatory ducts running from the bursa to the spermathecae (Coddington 1986a: figs 145, 152). Surface of epigynal plate is smooth and its posterior margin generally lacks scape-like structures. Shape of posterior margin of epigynal plate varies among *Theridiosoma* species: rounded or almost straight in some species, while having a pair of small, sclerotized processes (named as ‘spurs’ in Coddington 1986a) or a small slit-like invagination in others (Coddington 1986a; Miller et al. 2009; Suzuki et al. 2020). *Zoma* females possess a similar genitalia except in having a sclerotized median pit on the surface of the epigynal plate and lacking any processes nor invaginations on the posterior margin of the epigynal plate in known species (Saaristo 1996; Miller et al. 2009; Zhao and Li 2012; Ballarin et al. 2021).

#### ﻿ Theridiosoma nigrivirgatum sp. nov.

Taxon classificationAnimaliaAraneaeTheridiosomatidae

7DB5E535-0D4B-5187-80EE-D99D3A3E129C

https://zoobank.org/C1CB60C1-B782-4E34-9FAB-F44D3B12B4A0

**Figure 1.**
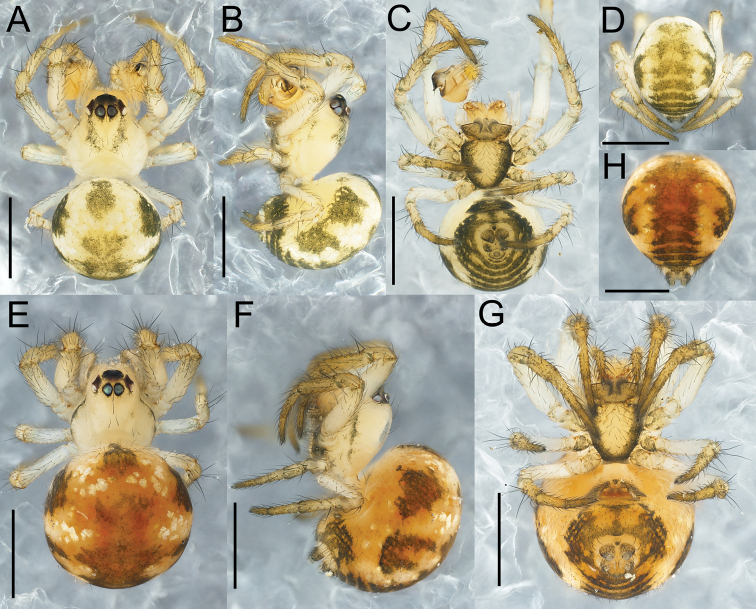
*Theridiosomanigrivirgatum* sp. nov., male holotype (NSMT-Ar 21717 **A–D**) and female paratype (NSMT-Ar 21718 **E–H**) **A, E** habitus, dorsal view **B, F** habitus, lateral view **C, G** habitus, ventral view **D, H** abdomen, posterior view. Scale bars: 0.5 mm.

**Figure 2.**
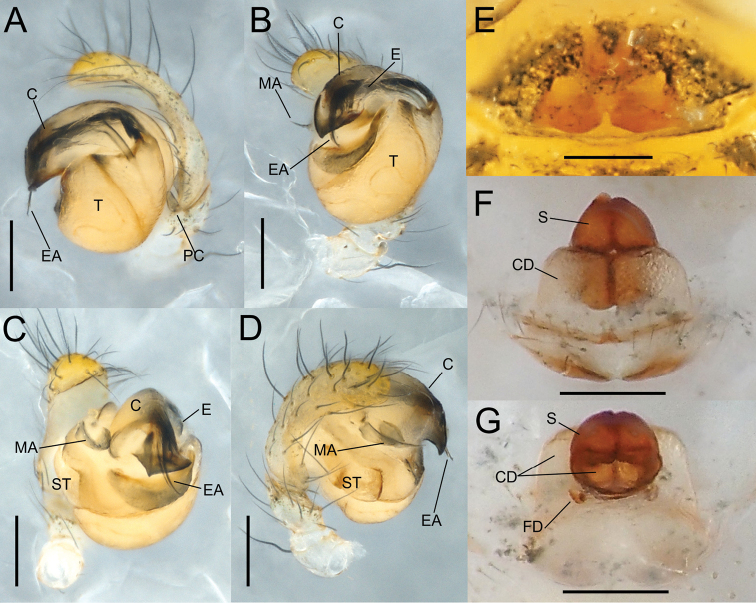
*Theridiosomanigrivirgatum* sp. nov., male holotype genitalia (NSMT-Ar 21717 **A–D**) and female paratype genitalia (NSMT-Ar 21718 **E–G**) **A** retrolateral view **B** ventral view **C** posterior-ventral view **D** prolateral view **E** ventral view **F** ventral view **G** dorsal view. Abbreviations: **C** conductor **CD** copulatory ducts **E** embolus **EA** embolic apophysis **FD** fertilization ducts **MA** median apophysis **PC** paracymbium **S** spermatheca **ST** subtegulum **T** tegulum. Scale bars: 0.1 mm.

**Figure 3.**
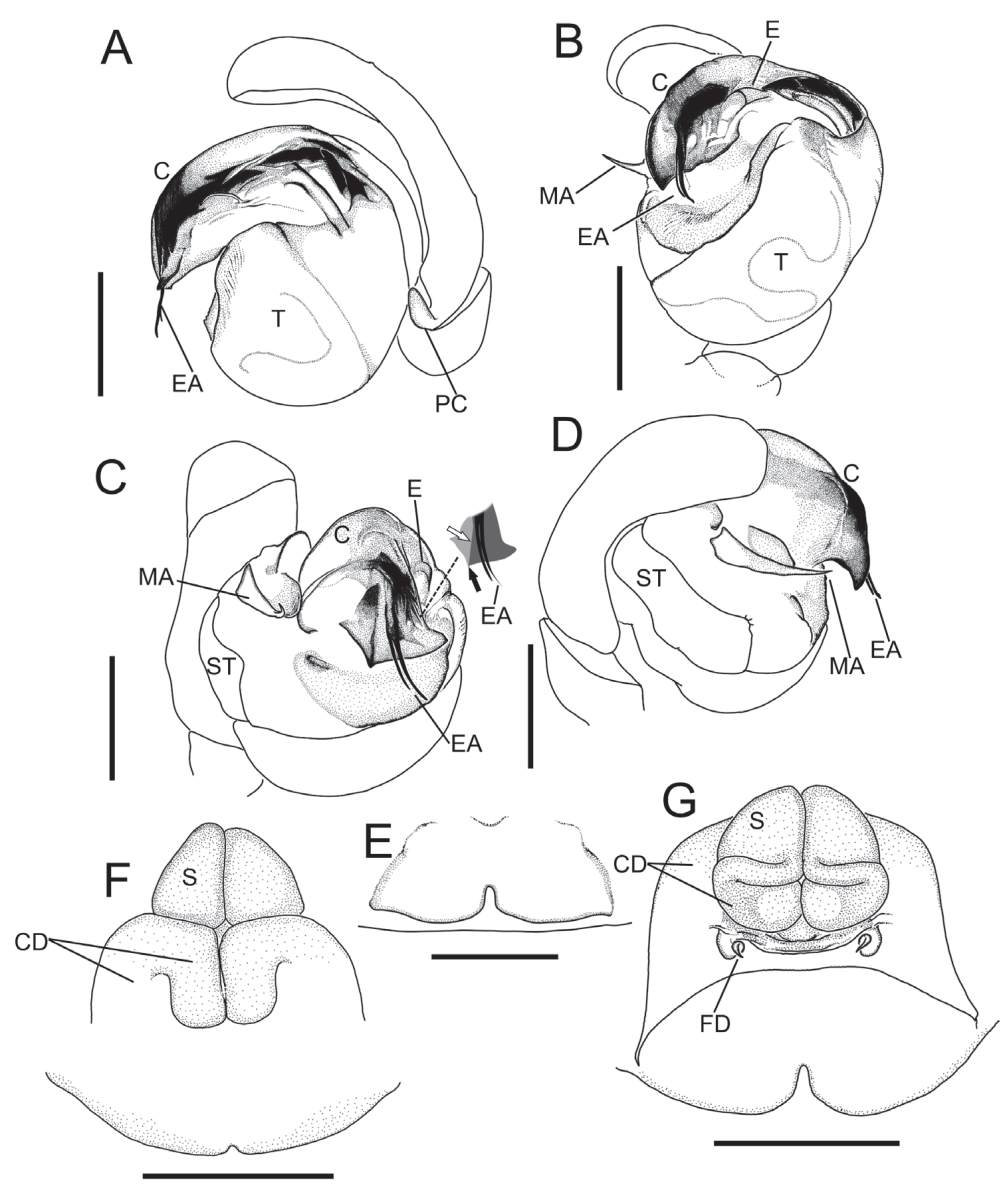
*Theridiosomanigrivirgatum* sp. nov., male holotype genitalia (NSMT-Ar 21717 **A–D**) and female paratype genitalia (NSMT-Ar 21718 **E–G**) **A** retrolateral view **B** ventral view **C** posterior-ventral view **D** prolateral view **E** ventral view **F** ventral view **G** dorsal view. Abbreviations: **C** conductor **CD** copulatory ducts **E** embolus **EA** embolic apophysis **FD** fertilization ducts **MA** median apophysis **PC** paracymbium **S** spermatheca **ST** subtegulum **T** tegulum. White and black arrows indicate a ridge that separate the two triangular surfaces and a sharply cornered terminal of the ridge, respectively. Scale bars: 0.1 mm.

[New Japanese name: Jyabara-karakara-gumo] Figs 1 , 2 , 3 , 11 , 12C , 13A–C , 15A

##### Type material.

***Holotype*: Japan, Okinawa Is. (Okinawa Prefecture)**: ♂ (NSMT-Ar 21717), Urasoe City, Nakama, Urasoe-daikoen Park (26°14'50.2"N, 127°43'49.8"E, alt. 112 m), 8 Mar. 2021, Y. Suzuki leg. ***Paratypes***: 2 ♀, same data as the holotype; 1 ♂ 1 ♀ (NSMT-Ar 21718), Urasoe City, Nakama, Urasoe-daikoen Park (26°14'59.2"N, 127°43'54.6"E, alt. 64 m), 16 Apr. 2021, Y. Suzuki leg.; 1 ♂ 1 ♀ (NSMT-Ar 21719), Nakagami District, Nishihara Town, Senbaru (26°15'01.8"N, 127°45'57.8"E, alt. 104 m), 25 Apr. 2021, Y. Suzuki leg.

##### Other material examined.

**Japan, Okinawa Is. (Okinawa Prefecture)**: 10 ♀, Naha City, Shuri-sueyoshi Town, Sueyoshi-koen Park (26°13'45.0"N, 127°42'49.8"E, alt. 49 m), [7 Mar. 2021 (1 ♀), 8 Mar. 2021 (9 ♀)], Y. Suzuki leg.; 5 ♀, Nakagami District, Nishihara Town, Tanabaru, Tanabaru Gusuku (26°14'44.3"N, 127°45'16.4"E, alt. 141 m), 8 Apr. 2021, Y. Suzuki leg.; 2 ♂, Kunigami District, Kunigami Village, Yona (26°44'35.2"N, 128°14'55.1"E, alt. 195 m), 19 Sep. 2021, Y. Suzuki leg.; 1 ♀, Ôgusuku (26°17'09.5"N, 127°48'13.1"E, alt. 136 m), Nakagami Distirct, Kitanakagusuku Village, R. Serita leg. **Kume Is. (Okinawa Prefecture)**: 1 ♀ 2 juv., Shimajiri District, Kumejima Town, Jyanado (26°20'46.2"N, 126°47'52.0"E, alt. 16 m), 10 Sep. 2021, Y. Suzuki leg. **Aka Is. (Okinawa Prefecture)**: 1 ♀, Shimajiri District, Zamami Village, Aka, streamside at dim forest (26°11'47.11"N, 127°16'57.09"E, alt. 65 m), 16 Mar. 2022, Y. Suzuki leg.

##### Etymology.

The specific name is a Latin adjective derived from the black striped pattern on the dorsal abdomen of the new species.

##### Diagnosis.

Males of the new species resemble *T.alboannulatum* Suzuki, Serita & Hiramatsu, 2020 in having two parallel embolic apophyses exposed from conductor and lacking a conductor projection on the male palp. They can be distinguished by the presence of one embolic apophysis longer than another and the shape of the sclerotized distal margin of conductor beneath embolic apophyses: a ridge separates the two triangular surfaces and sharply cornered at the terminal of the ridge in *T.nigrivirgatum* sp. nov. (Figs 2C, 3C), while a ridge is lacking in *T.alboannulatum* (Suzuki et al. 2020: fig. 12E, F). Females of the new species resemble those of *T.diwang* Miller, Griswold & Yin, 2009 in having a small and narrow slit on the posterior margin of the epigynal plate, but can be distinguished by the shape of the vulva: genital plate is bell-shaped and longer than wide; spermathecae are positioned at the anterior part of the vulva in *T.nigrivirgatum* sp. nov. (Figs 2G, 3G), while the vulva is wider than long, copulatory ducts extend anteriorly, and the position of spermatheca is lower than the anterior margin of the copulatory ducts in *T.diwang* (Miller et al. 2009: fig. 3G). Both sexes can be distinguished from congeners by their abdominal color and patterns: a dark marking on the anterior dorsum, two pairs of dark markings on the dorsolateral side, and dark striped markings on the posterior dorsum (Fig. 1).

##### Description.

**Male** (holotype, NSMT-Ar 21717). Measurements. Body 1.02 long. Carapace 0.45 long, 0.46 wide, and 0.36 high. Eye size and interdistances, AME 0.054, ALE 0.047, PME 0.050, PLE 0.042, AME-AME 0.022, AME-ALE 0.017, PME-PME 0.012, PLE-PLE 0.030. Leg length: leg I 0.47 + 0.17 + 0.31 + 0.29 + 0.20 = 1.44; leg II 0.38 + 0.15 + 0.26 + 0.24 + 0.18 = 1.21; leg III 0.23 + 0.13 + 0.14 + 0.18 + 0.13 = 0.81; leg IV 0.30 + 0.13 + 0.20 + 0.20 + 0.15 = 0.98. Abdomen 0.58 long, 0.60 wide, 0.80 high.

Carapace oval, wider than long (CaL/CaW 0.98). Chelicerae with three teeth on promargin. Abdomen oval and wider than long (AL/AW 0.97).

Coloration and markings (Fig. 1A–D). Carapace, chelicerae, and legs dark yellowish brown (turning to yellowish brown in ethanol). Cephalic groove stained with dark spots. Anterolateral margin of carapace dark grey. Mouthparts dark yellowish brown. Sternum pale yellowish brown with black lateral margins. Eyes on the dark bases. Legs yellowish brown with femora pale and lacking annulations. Abdomen pale yellowish brown with a dark greyish marking on anterior dorsum, two pairs of dark greyish spots on dorsolateral sides, and dark-colored longitudinal stripes on posterior dorsum. Spinnerets and ventral side of abdomen dark grey.

Palp (Figs 2A–D, 3A–D). Palpal patella with a strong retrolateral macroseta. Paracymbium hook-like with a blunt tip. Tegulum bulbous. Embolic division covered with a semitransparent conductor and composed of several apophyses. Conductor lacking conductor projection. Two long and parallel bristle-like embolic apophyses exposed from the conductor. Posterior margin of the embolic division strongly sclerotized with angular corners, the middle one pointed, the retrolateral one blunt, a ridge separates two triangular surfaces: one is covered by embolic division and the other is not (Fig. 3C). Tegular surface beneath conductor weakly sclerotized with denticles. Median apophysis narrower toward the pointed tip.

**Female** (paratype: NSMT-Ar 21718). Measurements. Body 1.31 long. Carapace 0.51 long, 0.50 wide, 0.41 high. Eye size and interdistances: AME 0.057, ALE 0.058, PME 0.060, PLE 0.053, AME-AME 0.019, AME-ALE 0.032, PME-PME 0.009, PLE-PLE 0.043. Leg length: leg I 0.61 + 0.20 + 0.29 + 0.26 + 0.19 = 1.55; leg II 0.40 + 0.16 + 0.24 + 0.19 + 0.16 = 1.15; leg III 0.24 + 0.15 + 0.14 + 0.17 + 0.13 = 0.83; leg IV 0.40 + 0.17 + 0.23 + 0.20 + 0.14 = 1.97. Abdomen 0.86 long, 0.87 wide, 0.90 high.

Carapace oval and almost as long as wide (CaL/CaW 1.02). Chelicerae with three teeth on promargin. Abdomen oval and as long as wide (CaL/CaW 0.99).

Coloration and markings (Fig. 1E–H). Carapace and chelicerae pale yellowish brown. Lateral margin of carapace dark grey. Eyes on the dark bases. Eyes, mouthparts, sternum, and legs as in male. Abdomen yellowish brown with dark greyish markings similar to the male, and dark orange markings on the dorsum and sides.

Genitalia (Figs 2E–G, 3E–G). Epigyne a wide plate with a short and narrow slit in the middle of the posterior margin. Vulva. Copulatory ducts moderately complicated. Spermathecae rounded triangular and juxtaposed. Fertilization ducts with curved tips.

Variations. The color and patterns of the abdomen vary: male specimens collected from Northern Okinawa lack longitudinal stripes on the posterior dorsum of the abdomen.

##### Taxonomic justification.

*Theridiosomanigrivirgatum* sp. nov. can safely be assigned to the genus according to the male palpal morphology: embolus short and tubular, and embolus apophyses fragmented into several long bristle-like parts.

##### Remarks.

The males and females are considered to be the same species because of the similarity of body color and patterns and their sympatric occurrences. Although this species sympatrically occurred with *T.dissimulatum* on southern Okinawa Island (Fig. 11), no other undescribed candidates were collected.

##### Distribution.

Japan (Okinawa, Kume and Aka Islands; Fig. 11).

##### Habitat.

The new species inhabits forest floors of secondary forests, bushes, and grasslands. The species is frequently collected from an open environment covered by Poaceae grasses, where *T.dissimulatum* is never found (Fig. 12C). The habitat of this species resembles that of *T.alboannulatum* (Fig. 12D).

##### Web morphology.

This species weaves a concave orb web with radial anastomosis and a tension line connected to substrates (Fig. 13A–C). A spider drags a tension line with a strong force so that the web is deformed to a conical shape. The web is similar to that of the congeners.

##### Egg sac.

pale whitish brown and spherical with a long horizontal line and a short stalk (Fig. 15A).

#### ﻿ Theridiosoma dissimulatum

Taxon classificationAnimaliaAraneaeTheridiosomatidae

Suzuki, Serita & Hiramatsu, 2020

6441B783-04AC-5134-86B8-03D21C6660E5

Figs 11 , 12A , 15B

Theridiosoma dissimulatum Suzuki, Serita & Hiramatsu, 2020: 137, figs 1E–H, 3D–F, 5C, D, 8A–J, 9A–P, 13E–H (holotype male and paratypes from Amami Island, Japan; not examined).

##### Material examined.

**Japan, Amami Is. (Kagoshima Prefecture)**: 1 ♀, Amami City, Nase-uragami Town (28°23'55.6"N, 129°32'27.5"E, alt. 139 m), 1 Jul. 2021, Y. Suzuki leg.; 4 ♀, Amami City, Sumiyo Town, Nishinakama, Santaro-toge Pass (28°15'48.7"N, 129°25'09.0"E, alt. 141 m), 6 May 2021, Y. Suzuki leg.; 1 ♀, Ôshima District, Yamato Village, Ôganeku, Materiya-no-taki Waterfall (28°19'04.4"N, 129°21'08.2"E, alt. 176 m), 4 Jul. 2021, Y. Suzuki leg. **Okinoerabu Is. (Kagoshima Prefecture)**: 2 ♂ 4 ♀, Ôshima District, China Town, Tokudoki (27°21'34.5"N, 128°33'02.8"E, alt. 134 m), 8 Dec. 2021, Y. Suzuki leg. **Okinawa Is. (Okinawa Prefecture)**: 2 ♂ 3 ♀, Naha City, Shuri-sueyoshi Town, Sueyoshi-koen Park (26°13'39.6"N, 127°42'55.3"E, alt. 25 m), 7 Mar. 2021, Y. Suzuki leg.; 2 ♀, Kunigami District, Ôgimi Village, Nerome (26°40'49.7"N, 128°08'01.1"E, alt. 128 m), 14 Apr. 2021, Y. Suzuki leg.; 1 ♀, Kunigami District, Ôgimi Village, Ôgimi (26°40'57.9"N, 128°08'21.6"E, alt. 311 m), 15 May 2021, Y. Suzuki leg. **Iriomote Is. (Okinawa Prefecture)**: 1 ♂ 3 ♀, Yaeyama District, Taketomi Town, Haiminaka, Ôtomi-rindo Path (24°17'52.6"N, 123°52'47.3"E, alt. 18 m), 30 Apr. 2021, Y. Suzuki leg.; 1 ♂ 3 ♀, Ôtomi-daiichi-do Cave (24°17'31.0"N, 123°52'45.7"E, alt. 30 m), 1 May 2021, Y. Suzuki leg.

##### Remarks.

This species can easily be distinguished from *T.nigrivirgatum* sp. nov. by the presence of a conductor projection on the male palp and a heart-shaped invagination with a pair of spurs on the posterior margin of the female epigynal plate (Suzuki et al. 2020). Refer to the description in Suzuki et al. (2020) for further morphological information.

##### Distribution.

Japan (Amami, Okinoerabu, Okinawa, Ishigaki, and Iriomote Islands; Fig. 11).

##### Habitat.

This species was collected from dim moist forests, especially from locations beside streams (Fig. 12A).

##### Web morphology.

*Theridiosomadissimulatum* weaves a concave orb web and drags a tension line with the forelegs.

##### Egg sac.

pale reddish brown and spherical with a long horizontal line and a short stalk (Fig. 15B).

#### ﻿ Theridiosoma alboannulatum

Taxon classificationAnimaliaAraneaeTheridiosomatidae

Suzuki, Serita & Hiramatsu, 2020

ED480740-B043-5179-884C-A5007623E190

Figs 12D , 13D–E , 15C

Theridiosoma alboannulatum Suzuki, Serita & Hiramatsu, 2020: 149, figs 2I–L, 4G–I, 6E–F, 12A–J, 13P (holotype male and paratypes from Iriomote Island, Japan; not examined).

##### Material examined.

**Japan, Kurima Is. (Okinawa Prefecture)**: 2 ♂, Miyakojima City, Shimojikuruma (24°43'29.2"N, 125°15'09.2"E, alt. 41 m),, 17 Nov. 2021, Y. Suzuki leg. **Miyako Is. (Okinawa Prefecture)**: 3♂ 3 ♀, Miyakojima City, Hiraranishihara, grassland at roadside (24°49'53.5"N, 125°18'55.0"E, alt. 46 m), 24 Apr. 2022, Y. Suzuki leg. **Kuroshima Is. (Okinawa Prefecture)**: 2 ♂ 1♀, Yaeyama District, Taketomi Town, edge of coastal forest besides Hokei beach (24°14'26.2"N, 123°59'32.7"E, alt. 0 m), 2 Nov. 2021, Y. Suzuki leg. **Yonaguni Is. (Okinawa Prefecture)**: 1 ♀, Yaeyama District, Yonaguni Town, Yonaguni (24°27'58.8"N, 123°01'20.1"E, alt. 49 m), 12 Oct. 2021, Y. Suzuki leg.; 2 ♂ 2♀, Yaeyama District, Yonaguni Town, Sonai Village (24°28'10.5"N, 123°00'31.7"E, alt. 19 m), 12 Oct. 2021, Y. Suzuki leg.; 1 ♂ 1♀, Yaeyama District, Yonaguni Town, wetland beside secondary forest (24°27'16.3"N, 122°59'24.3"E, alt. 38m), 14 Oct. 2021, Y. Suzuki leg.

##### Note.

See diagnosis section for comparison with *T.nigrivirgatum* sp. nov.

##### Habitat.

This species inhabits grasslands, bushes, and secondary forests. Spiders were collected from the basal parts of grasses.

##### Web morphology.

The spider weaves a concave web between the grasses (Fig. 13D, E).

##### Egg sac.

similar to that of *T.nigrivirgatum* sp. nov. (Fig. 15C).

##### Distribution.

Japan (Miyako, Kurima, Iriomote, Kuroshima, and Yonaguni Islands; Fig. 11).

#### ﻿Genus Zoma

Taxon classificationAnimaliaAraneaeTheridiosomatidae

Saaristo, 1996

800441AD-22AB-5981-83A8-F860B8919AE2

##### Type species.

*Zomazoma* Saaristo, 1996, from Seychelles (not examined).

##### Composition.

*Zomazoma* Saaristo, 1996, *Z.dibaiyin* Miller, Griswold & Yin, 2009, *Z.fascia* Zhao & Li, 2012, *Z.taiwanica* (Zhan, Zhu & Tso, 2006).

##### Remarks.

**Figure 4.**
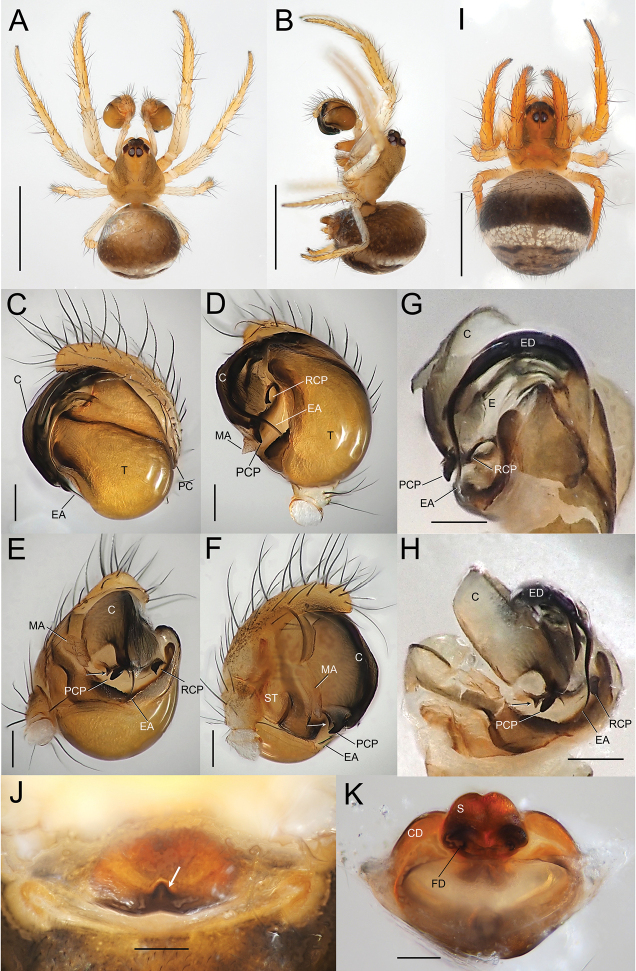
*Zomadibaiyin* Miller, Griswold & Yin, 2009, male habitus and genitalia (NSMT-Ar 21720 **A–H**) and female habitus and genitalia (NSMT-Ar 21721 **I–K**) **A** habitus, dorsal view **B** habitus, lateral view **C** palp, retrolateral view **D** palp, ventral view **E** palp, posterior-ventral view **F** palp, prolateral view **G** embolic division, prolateral view **H** embolic division, posterior-ventral view **I** habitus, dorsal view **J** epigyne, ventral view **K** vulva, dorsal view. Abbreviations: **C** conductor **CD** copulatory ducts **E** embolus **EA** embolic apophysis **FD** fertilization ducts **MA** median apophysis **PC** paracymbium **PCP** posterior conductor projection **RCP** retrolateral conductor projection **S** spermatheca **ST** subtegulum **T** tegulum. Arrows in **E, F, H** indicate a cornered margin of posterior membrane of conductor. Arrow in **J** indicates a sclerotized median pit. Scale bars: 1.0 mm (**A, B, I**); 0.1 mm (**C–H, J, K**).

**Figure 5.**
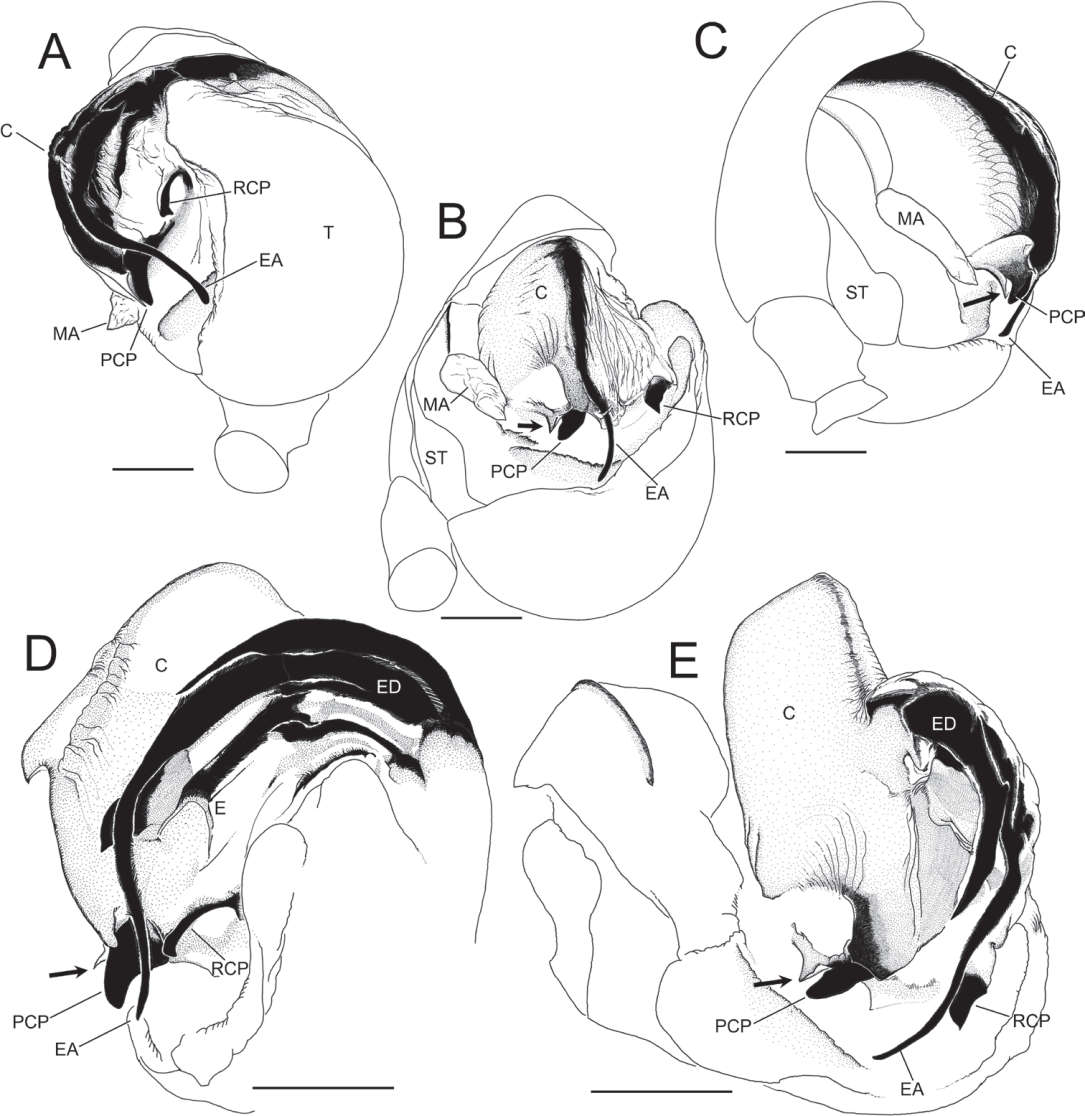
*Zomadibaiyin* Miller, Griswold & Yin, 2009, male genitalia (NSMT-Ar 21720) **A** ventral view **B** posterior-ventral view **C** prolateral view **D** conductor and embolic division, retrolateral view **E** conductor and embolic division, posterior-ventral view. Abbreviations: **C** conductor **CD** copulatory ducts **E** embolus **EA** embolic apophysis **MA** median apophysis **PC** paracymbium **PCP** posterior conductor projection **RCP** retrolateral conductor projection **ST** subtegulum **T** tegulum. Arrows in **B–E** indicate a cornered margin of posterior membrane of conductor. Scale bars: 0.1 mm.

Females of the genus can be distinguished by the flat and bluntly triangular genital plate with a sclerotized median pit and a pair of smaller, generally less recognizable, lateral pits (Fig. 4J; see also Saaristo 1996). Males of the type species *Z.zoma* have not yet been described. Therefore, the taxonomic characteristics of *Zoma* males are poorly defined. Males of three *Zoma* species, *Z.dibaiyin*, *Z.fascia*, and *Z.taiwanica* have relatively simpler palps with a filiform embolic apophysis emerging beneath from the conductor, while two or more apophyses in *Theridiosoma* (Fig. 3B, C vs. Fig. 5A, B). *Zoma* species have wider and straight median apophysis, while curved and sharp tip in *Theridiosoma* (Fig. 3B–D vs. Fig. 5A–C). *Zoma* species can be distinguished from congeners by the presence of a transverse whitish silver band on the dorsum abdomen (Saaristo 1996; Miller et al. 2009).

#### ﻿ Zoma dibaiyin

Taxon classificationAnimaliaAraneaeTheridiosomatidae

Miller, Griswold & Yin, 2009

ECB883B6-8020-5321-9478-AC0800388169

Figs 4 , 5 , 11 , 12B , 13F–H

Zoma dibaiyin Miller, Griswold & Yin, 2009: 27, figs 10A–F, 11A–B, 13A–D (holotype male and paratypes from China; not examined); Ono and Ogata 2018: 120, 504, 505; Suzuki and Serita 2021a: 231, figs 1–3.

##### Material examined.

**Japan, Amami Is. (Kagoshima Prefecture)**: 1♂ 1 ♀ (NSMT-Ar. 21720, 21721), Ôshima District, Setouchi Town, Katsuura (28°12'33.7"N, 129°19'54.0"E, alt. 354 m), 4 Jul. 2021, Y. Suzuki leg.; 1 ♂ 1 ♀, Amurogama (28°13'15.4"N, 129°18'59.3"E, alt. 111 m), 4 Jul. 2021, Y. Suzuki leg.; 1 ♂, Amami City, Sumiyo Town, Nishinakama, Santarou-toge Pass (28°15'48.7"N, 129°25'09.0"E, alt. 141 m), 6 May 2021, Y. Suzuki leg.; **Okinawa Is. (Okinawa Prefecture)**: 1 ♂ 1 ♀, Kunigami District, Ôgimi Village, Nerome (26°40'49.7"N, 128°08'01.1"E, alt. 128 m), 14 Apr. 2021, Y. Suzuki leg.; **Kume Is. (Okinawa Prefecture)**: 1 ♂ 1 ♀, Shimajiri District, Kumejima Town, Uezu, Mt. Daruma-yama (26°21'42.9"N, 126°45'34.9"E, alt. 149 m), 10 Sep. 2021, Y. Suzuki leg.

##### Diagnosis.

Males of this species can be distinguished from congeners by the embolic apophysis with a curved tip running along the sclerotized surface of the ventral tegulum, and females by a nearly transverse posterior margin of the epigynal plate (more convex in *Z.zoma* and rounded in *Z.taiwanica*) and lower position of the spermathecae (higher in *Z.taiwanica*) (Miller et al. 2009; Ballarin et al. 2021).

##### Description.

**Male** (NSMT-Ar 21720). Measurements. Body 1.62 long. Carapace 0.74 long, 0.68 wide, 0.60 high. Eye size and interdistances: AME 0.090, ALE 0.076, PME 0.093, PLE 0.065, AME-AME 0.015, AME-ALE 0.032, PME-PME 0.008, PLE-PLE 0.070. Leg length: leg I 0.80 + 0.23 + 0.57 + 0.47 + 0.28 = 2.35; leg II 0.50 + 0.21 + 0.46 + 0.31 + 0.28 = 1.76; leg III 0.40 + 0.19 + 0.23 + 0.24 + 0.24 = 1.30; leg IV 0.44 + 0.14 + 0.34 + 0.27 + 0.27 = 1.46. Abdomen 0.88 long, 0.95 wide, 1.01 high.

Carapace oval, longer than wide (CaL/CaW 1.34). Chelicerae with three teeth on promargin. Abdomen oval, wider than long (AL/AW 0.93).

Coloration and markings (Fig. 4A, B). Carapace, chelicerae, maxillae, labium, sternum, and legs yellowish brown. Eyes on the dark bases. Cephalic groove stained with dark spots. Legs lacking annulation. Abdomen dark brown encircled dorsolaterally with a whitish silver band.

Palp (Figs 4C–H, 5). Paracymbium with sharp tip. Tegulum bulbous. Median apophysis weakly sclerotized, wider than long. Embolic division branched into a few bristle-like apophyses, embolus short and tubular, and a long and filiform embolic apophysis emerging beneath from a translucent conductor. Tip of embolic apophysis curved and running along sclerotized surface of the tegulum beneath the conductor. Conductor having two projections: posterior conductor projection strongly sclerotized and triangular with a blunt tip; retrolateral conductor projection strongly sclerotized and weakly curved anteriorly with a triangular posterior tip. Posterior margin of conductor with a sharp tip.

**Female** (NSMT-Ar 21721). Measurements. Body 2.04 long. Carapace 0.82 long, 0.76 wide, 0.67 high. Eye size and interdistances: AME 0.089, ALE 0.082, PME 084, PLE 0.076, AME-AME 0.023, AME-ALE 0.034, PME-PME 0.009, PLE-PLE 0.076. Leg length: leg I 0.66 + 0.32 + 0.42 + 0.33 + 0.22 = 1.95; leg II 0.64 + 0.30 + 0.41 + 0.33 + 0.21 = 1.89; leg III 0.36 + 0.18 + 0.20 + 0.25 + 0.18 = 1.17; leg IV 0.53 + 0.21 + 0.34 + 0.27 + 0.22 = 1.57. Abdomen 1.21 long, 1.32 wide, 1.16 high.

Carapace, mouthparts, and abdomen as in male (CaL/CaW 1.08; AL/AW 0.92).

Coloration and markings (Fig. 4I) similar to male.

Genitalia (Fig. 4J, K). Epigynal plate flat and wider than long with a sclerotized posterior margin and a median pit. Spermathecae touching each other, copulatory ducts wide at their openings, and the course of the ducts simple. See Miller et al. (2009) for further details.

##### Remarks.

A strongly sclerotized triangular projection with a rounded tip (Figs 4E, 5B) and a cornered margin of the posterior membrane of the conductor (Figs 4E, 5B, arrowed) were visible in the ventro-posterior view of the male palp. The triangular projection does not seem to be homologous to conductor projection in *Theridiosoma*, as the former protrudes from the posterior margin of the conductor, while the latter was positioned on the surface of the conductor. Herein, we define it as posterior conductor projection (PCP).

The embolus on the male palp was not determined in *Z.dibaiyin* and *Z.fascia* (Miller et al. 2009; Zhao and Li 2012). In *Z.taiwanica*, the embolus is described as a ‘short and tubular structure’ but lacks explanations in illustrations (Zhang et al. 2006). Ballarin et al. (2021) determined the embolus as a strongly sclerotized, thin, stick-like apophysis located on the retrolateral side of the embolic division (Ballarin et al. 2021). Considering the shape of *Theridiosoma*’s embolus, which is short, tubular, and hidden under conductor (see Coddington 1986a: fig. 131), we suppose the ‘embolus’ of *Zoma* species determined in Ballarin et al. (2021) is not an embolus. The true embolus of *Z.dibaiyin* is found beneath basal part of embolic apophyses (Fig. 5D). Hereafter we defined the sclerotized stick-like structure as a retrolateral conductor projection (RCP), and distinguishable from an embolus. The shape of RCP is useful as a taxonomic character for differentiating species within the genus *Zoma*: S-shaped with pointed tip in *Z.fascia*, claviform with rounded tip in *Z.taiwanica* (Zhao and Li 2012: figs 28, 30; Zhang et al. 2006: figs 4–6; Ballarin et al. 2021: fig. 5B, C), and weakly curved anteriorly with a triangular posterior tip in *Z.dibaiyin* (Figs 4D, 5D).

##### Distribution.

China (Yunnan), Japan (Honshu to the Ryukyu Islands; Fig. 11).

##### Habitat.

This species inhabits the forest floor and streamside in dim and wet forests (Fig. 12B).

##### Web morphology.

This species weaves a concave orb web with radial anastomosis above the ground (Fig. 13F–H). The orb web is almost horizontal. A tension line stretched from the center of the web and attached to substrates such as rocks and dead leaves. The mesh of sticky spirals tends to be fine (occasionally, the number of sticky lines is > 30). For details of the web morphology, see Hiramatsu (2021).

##### Egg sac.

light brown with a distinct circular suture at the upper end. The sac was suspended from a long horizontal line with a short stalk (see Hiramatsu 2021).

#### ﻿Genus Sennin gen. nov.

Taxon classificationAnimaliaAraneaeTheridiosomatidae

FCA223EB-749E-558D-B6A3-71A9D9C1B65B

https://zoobank.org/AAA86579-ABA5-4385-B0D7-68D7676BD871

##### Type species.

*Sennintanikawai* sp. nov.

##### Etymology.

The generic name *Sennin* is noun in apposition, masculine, and derived from the Japanese word meaning mountain hermits, a person who acquires a spiritual power after living a secluded life deep in the mountains. Iriomote Island, where the new species inhabits, is famous for a man called Sennin, who was self-sufficient, lived in the coastal caves, and single-handedly built a wooden hut.

##### Diagnosis.

This genus can be distinguished from other theridiosomatid genera by the following characteristics: a large, oblong cymbial outgrowth (cymbial apophysis) protruding from the basal and dorsal part of cymbium of male palp (Figs 7A–C, 9 A–C); an embolic division with three elongated bristle-like embolic apophyses with the longest one coiled (Figs 7F–J, 9F–J); the anterior margin of the epigynal plate with a pair of sclerotized, triangular extensions protruding anteriorly from the anterolateral side (Figs 8A, D; 10A, D; arrowed; Zhu et al. 2001: fig. 4; Chen 2010: figs 19, 20); the vulva with long copulatory ducts coiling at the lateral side of the spermatheca (Figs 8C, D, 10C, D).

**Figure 6.**
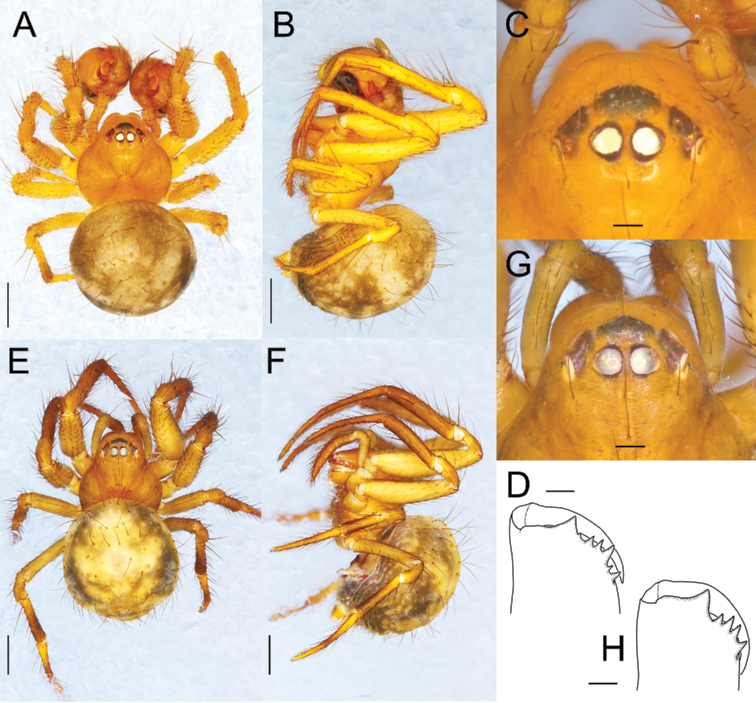
*Sennintanikawai* sp. nov., male holotype (NSMT-Ar 21722 **A–D**) and female paratype (NSMT-Ar 21723 **E–H**) **A, E** habitus, dorsal view **B, F** habitus, lateral view **C, G** eye region, dorsal view **D, H** chelicera, anterior view. Scale bars: 0.5 mm (**A, B, E, F**); 0.1 mm (**C, G, D, H**).

##### Composition.

*Sennintanikawai* sp. nov., *S.coddingtoni* (Zhu, Zhang & Chen, 2001), comb. nov.

##### Remarks.

**Figure 7.**
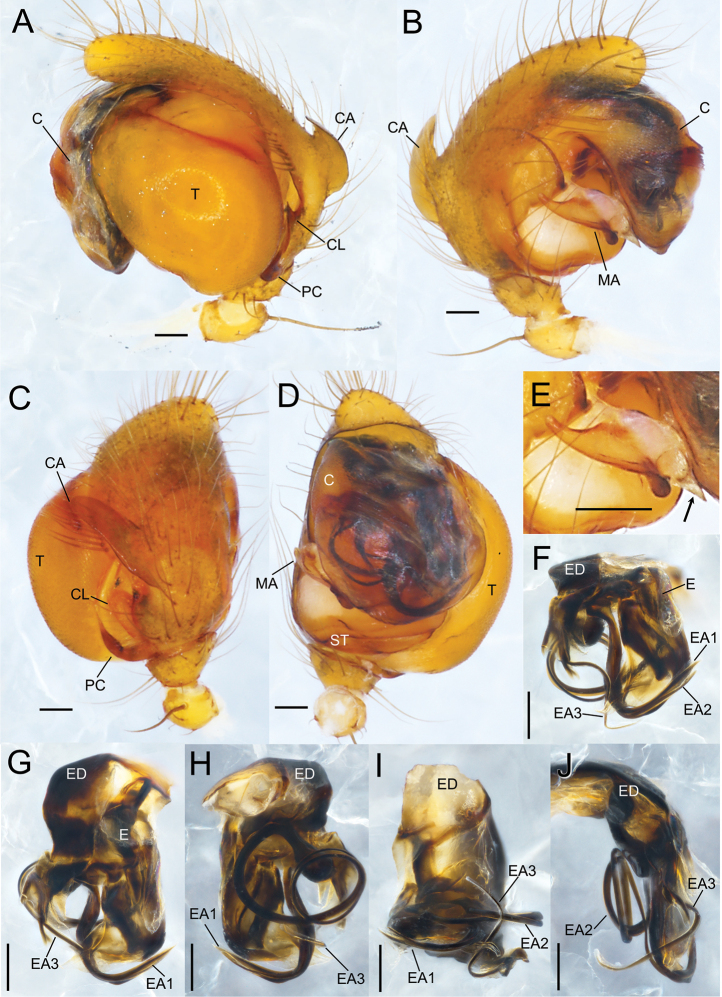
*Sennintanikawai* sp. nov., male holotype genitalia (NSMT-Ar 21722) **A–D** male palp **F–J** embolic division **A** retrolateral view **B** prolateral view **C** dorsal view **D** ventral view **E** median apophysis, ventral view **F** posterior-ventral view **G** ventral view **H** anterior-dorsal view **I** posterior-dorsal view **J** prolateral view. Abbreviations: **C** conductor **CA** cymbial apophysis **CL** cymbial lamella **E** embolus **EA** embolic apophysis **ED** embolic division **EM** embolus **MA** median apophysis **PC** paracymbium **ST** subtegulum **T** tegulum. Arrow in **E** indicates the tip of less-sclerotized region of median apophysis. Scale bars: 0.1 mm.

**Figure 8.**
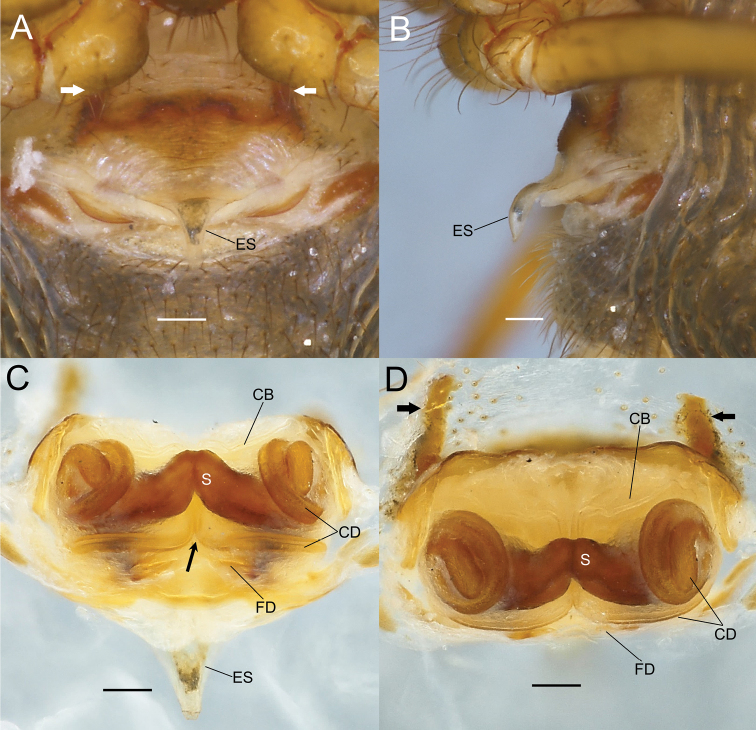
*Sennintanikawai* sp. nov., female paratype genitalia (NSMT-Ar 21723) **A** epigyne, ventral view **B** epigyne, lateral view **C** vulva, dorsal view **D** vulva, anterior view. Abbreviations: **CB** copulatory bursae **CD** copulatory ducts **ES** epigynal scape **FD** fertilization ducts **S** spermatheca. Arrows in **A, D** indicate a pair of sclerotized extensions on the anterior margin of epigynal plate. Arrow in **C** indicates a pair of copulatory ducts juxtaposed. Scale bars: 0. 1 mm.

**Figure 9.**
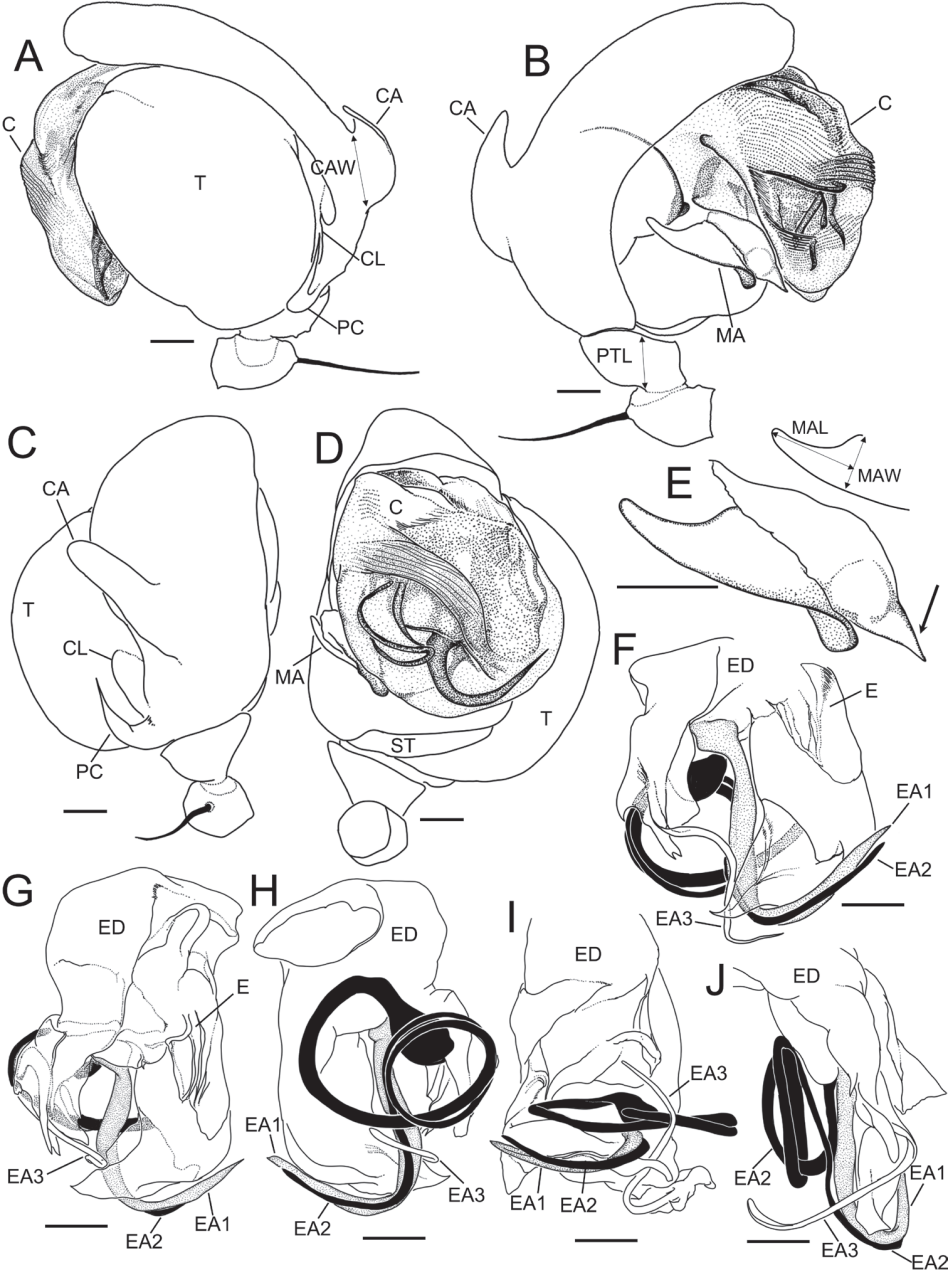
*Sennintanikawai* sp. nov., male holotype genitalia (NSMT-Ar 21722) **A–D** male palp **F–J** embolic division **A** retrolateral view **B** prolateral view **C** dorsal view **D** ventral view **E** median apophysis, ventral view **F** posterior-ventral view **G** ventral view **H** anterior-dorsal view **I** posterior-dorsal view **J** prolateral view. Abbreviations: **C** conductor **CA** cymbial apophysis **CL** cymbial lamella **E** embolus **EA** embolic apophysis **ED** embolic division **EM** embolus **MA** median apophysis **PC** paracymbium **ST** subtegulum **T** tegulum. Arrow in **E** indicates the tip of less-sclerotized region of median apophysis. Scale bars: 0.1 mm.

**Figure 10.**
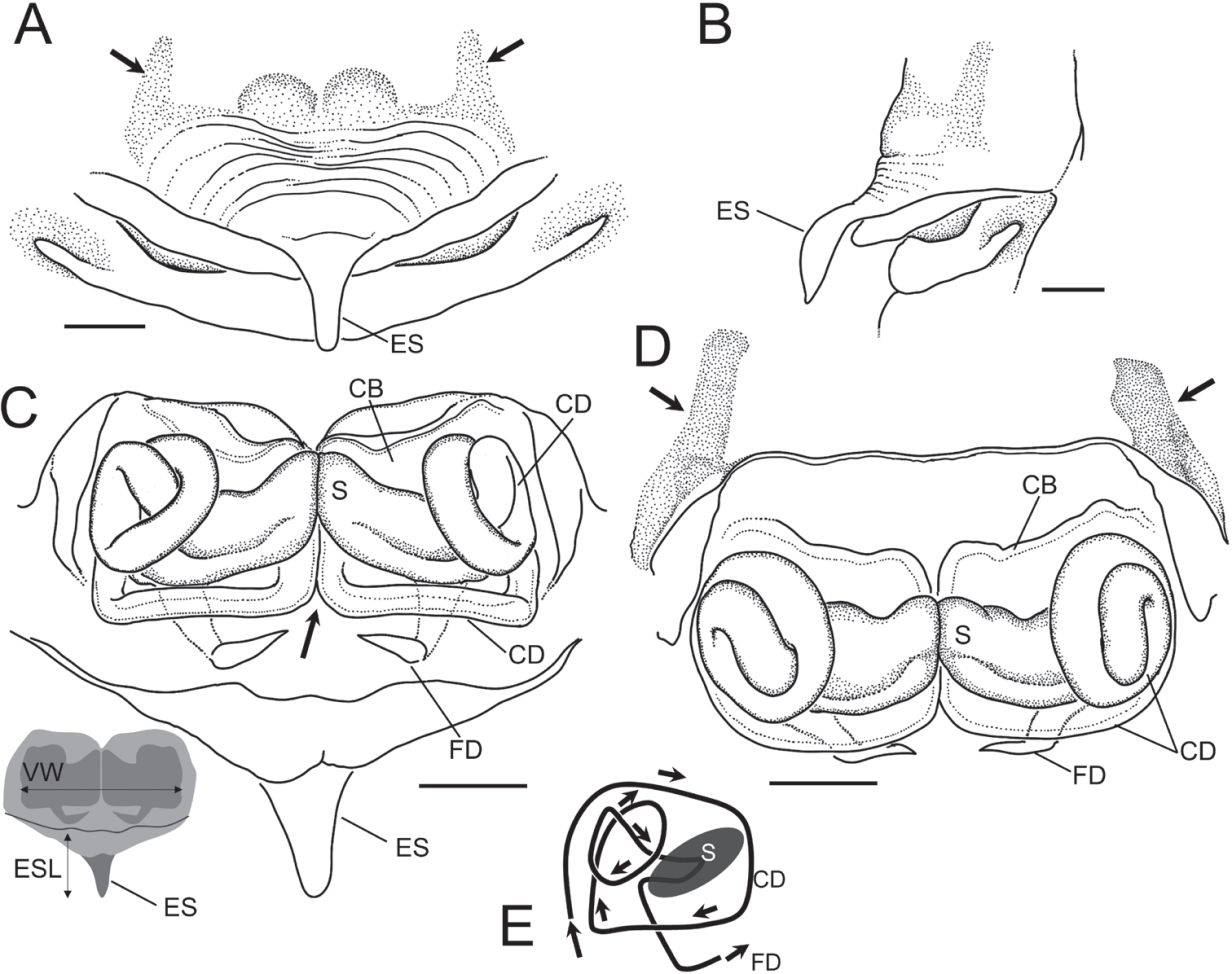
*Sennintanikawai* sp. nov., female paratype (NSMT-Ar 21723) **A** epigyne, ventral view **B** epigyne, lateral view **C** vulva, dorsal view **D** vulva, anterior view **E** course of copulatory duct. Abbreviations: **CB** copulatory bursae **CD** copulatory ducts **ES** epigynal scape **ESL** epigynal scape length **FD** fertilization ducts **S** spermatheca **VW** vulva width. Arrows in **A, D** indicate a pair of sclerotized extensions on the anterior margin of epigynal plate. Arrow in **C** indicates the pair of copulatory ducts juxtaposed. Scale bars: 0.1 mm.

This genus is related to *Baalzebub* Coddington, 1986, based on the shape of the median apophysis on the male palp, the embolic apophyses that are not exposed from the conductor, and the general morphology of the epigyne. The elongated and oblong dorsal cymbial apophysis, one of the most conspicuous characters of *Sennin* gen. nov. (Figs 7A–C, 9A–C), differentiates the new genus from *Baalzebub*. Although some species of *Baalzebub* have a small protrusion on the retrolateral-dorsal side of basal part of cymbium (e.g., paracymbium in *B.acutum*; Prete et al. 2016: figs 2D, 3C; named ‘Höcker’ (= lump) in *B.brauni*; Wunderlich 1976: figs 17, 18), it is not as prominent as that of *Sennin* gen. nov. The embolic apophyses of *Baalzebub* are short, blunt, and spatulate, but those of *Sennin* are longer, bristle-like, and strongly curved or coiled (Figs 7F–J, 9F–J). As for the female genitalia of species of *Baalzebub*, the epigynal plate is upside-down triangular with sclerotized central epigynal pit, the spermathecae elliptical, and longer laterally with connate tips, and the course of copulatory ducts is simple (Coddington 1986a). *Sennin* gen. nov. has similar spermathecae, but the course of the copulatory duct is more complex, with a coiled trajectory at the basal side of the spermathecae (Figs 8C, D, 10C, D).

*Sennincoddingtoni* comb. nov. was formerly placed in *Karstia* Chen, 2010, but it shares conspicuous characteristics with *S.tanikawai* sp. nov. and can clearly be differentiated from *K.upperyangtzica* Chen, 2010, the type species of the genus. Therefore, we transferred it from *Karstia* to *Sennin* gen. nov. *Karstiaupperyangtzica* and *K.cordata* Dou & Li (2012) females have an upside-down triangular epigynal plate with a sclerotized epigynal pit, and a simple course of copulatory ducts; males have cymbial apophysis as a very small protrusion, and embolic division with short, spatulate embolic apophyses (Chen 2010; Dou and Lin 2012; Zhang and Wang 2017). Based on these morphological characteristics, it is difficult to differentiate *K.upperyangtzica* and *K.cordata* from *Baalzebub*; therefore, taxonomic revision of *Karstia* is needed. In this study, we defer revision of *Karstia*, which may require direct examination of the type specimens and further molecular analysis.

As mentioned above, taxonomic relationship between *Sennin* gen. nov. and its potentially closest-related genera (*Baalzebub* and probably *Karstia*) is not yet well defined. This also indicated that the establishment of *Sennin* gen. nov. could render these related genera polyphyletic. To revise taxonomic status of these taxa in terms of monophyly, further integrative phylogenetic approach covering large number of species and genera is required.

According to the morphology and a potential close-relatedness to *Baalzebub*, *Sennin* gen. nov. is here suggested to be assigned to the subfamily Theridiosomatinae.

#### ﻿ Sennin tanikawai sp. nov.

Taxon classificationAnimaliaAraneaeTheridiosomatidae

03ED24B0-DB99-5094-84F5-D805F6BACC44

https://zoobank.org/2FB64512-C697-4195-AE82-62C4563508DD

[New Japanese name: Iriomote-hora-ana-karakara-gumo] Figs 6 , 7 , 8 , 9 , 10 , 11 , 12E , 14A–G , 15D–E

##### Type material.

***Holotype*: Japan, Iriomote Is. (Okinawa Prefecture)**: ♂ (NSMT-Ar 21722), Yaeyama District, Taketomi Town, Haiminaka, Ôtomi-Daini-Do Cave, 31 Mar. 1985, A. Tanikawa leg. ***Paratypes***: 2 ♀ (NSMT-Ar 21723), 27 Mar. 1995, A. Tanikawa leg.; 9 ♂ (NSMT-Ar 21724–21725), 31 Mar. 1985, A. Tanikawa leg.; 1 ♀ (NSMT-Ar 21726), 1 Aug. 1970, Y. Shirota leg.; 2 ♀ (NSMT-Ar 21727), 27 Oct. 1977, N. Tsurusaki leg.; above paratypes are collected at same locality as the holotype; 1 ♂ (NSMT-Ar 21728), a small opening of Ôtomi-Daini-Do Cave (24°17'09.4"N, 123°53'24.9"E, alt. 10 m), 24 Jun. 2021, Y. Suzuki leg.

##### Additional material examined.

**Japan, Iriomote Is. (Okinawa Prefecture)**: 1 ♀, Yaeyama District, Taketomi Town, Haemi, Ôtomi-daiichi-do Caves (24°17'31.0"N, 123°52'45.7"E, alt. 30 m), 3 May 2021, Y. Suzuki leg.; 1 ♀, Yaeyama District, Taketomi Town, Takana, Yutsun-Do Caves, a small cave on coastal cliff (24°23'08.90"N, 123°53'27.89"E, alt. 10 m), 21 Mar. 2019, Y. Suzuki leg.; 1 ♀, Takana, Yutsun-Do Caves, a large cave opening on coastal cliff (24°23'05.88"N, 123°53'25.00"E, alt. 7 m), 28 Mar. 2008, T. Hiramatsu leg.; 6 ♂ 7 ♀, Takana, Yutsun-Do Caves, a large cave opening on coastal cliff (24°23'05.88"N, 123°53'25.00"E, alt. 7 m), 1 May 2021, Y. Suzuki leg.; 2 ♀, Takana, Yutsun-Do Caves, cavities of rocks on coastal cliff (24°23'04.96"N, 123°53'23.82"E, alt. 10m), 22 Jun. 2021, Y. Suzuki leg.; 3 ♀, Takana, Yutsun-Do Caves, a cave beside Shirahama-haemi-sen road (24°23'06.5"N, 123°53'31.2"E, alt. 27 m), 24 Jun. 2021, Y. Suzuki leg.; 1 ♂ 1♀, Haemi, limestone rocky walls in a secondary forest (24°16'08.48"N, 123°52'01.52"E, alt. 16 m), 24 Jun. 2021, Y. Suzuki leg.

##### Etymology.

The specific name is patronym dedicated to Dr. Akio Tanikawa, a Japanese arachnologist who has contributed remarkably to the elucidation of the spider fauna in Iriomote Island and offered us many specimens including type specimens.

##### Diagnosis.

Males of this species can be distinguished from the allied *Sennincoddingtoni* comb. nov. by the following characteristics: cymbial apophysis is wider in relation to palpal tibia length while it is almost the same length as *S.coddingtoni* comb. nov. (CAW/PTL = 2.41 in *S.tanikawai* sp. nov., also see Fig. 9A, B; 1.00 in *S.coddingtoni* comb. nov.; also see Zhu et al. 2001: fig. 7); median apophysis of *S.tanikawai* sp. nov. is longer and narrower dorsally (MAL/MAW 1.63; Fig. 9E) compared to that of the latter (MAL/MAW 0.85, based on Chen 2010: fig. 24); the less-sclerotized distal part of median apophysis is lanceolate with pointed tip on ventral terminal in *S.tanikawai* sp. nov. (arrows in Figs 7E, 9E), while that of *S.coddingtoni* comb. nov. is falcate (Chen 2010: fig. 24). Females of *S.tanikawai* sp. nov. can be distinguished from *S.coddingtoni* comb. nov. by the following characteristics: longer epigynal scape (ESL/VW 0.46 in *S.tanikawai* sp. nov., Fig. 10; 0.16 in *S.coddingtoni* comb. nov., based on Chen 2010: fig. 19); tip of spermatheca is strongly curved anteriorly in *S.tanikawai* sp. nov., whereas it is almost straight in *S.coddingtoni* comb. nov.; the course of copulatory ducts: ducts from both sides juxtaposed at the middle of vulva ventral to spermathecae and continue posteriorly straight toward epigynal scape, then make a right-angle turn and apart laterally (arrows in Figs 8C, 10C), while in *S.coddingtoni* comb. nov. the ducts apart to each other ventrally to the spermatheca and curved laterally (Zhu et al. 2001: fig. 5).

##### Description.

**Male** (NSMT-Ar 21722). Measurements. Body 2.30 long. Carapace 1.07 long, 1.10 wide, 0.72 high. Eye size and interdistances: AME 0.09, ALE 0.09, PME 0.10, PLE 0.08, AME-AME 0.02, AME-ALE 0.03, PME-PME 0.03, PLE-PLE 0.08, Leg length: leg I 1.62 + 0.53 + 1.30 + 1.08 + 0.50 = 5.03; leg II 1.30 + 0.49 + 1.03 + 0.91 + 0.49 = 4.22; leg III 0.70 + 0.39 + 0.56 + 0.63 + 0.38 = 2.66; leg IV 0.93 + 0.40 + 0.73 + 0.69 + 0.38 = 3.14. Abdomen 1.32 long, 1.44 wide, 1.61 high.

Carapace oval, wider than long (CaL/CaW 0.97). Chelicerae with six teeth on promargin with the largest one positioned close to the fang base, no teeth on retromargin (Fig. 6D). Anterior eye row recurved, posterior eye row straight. Cymbial apophysis of palp 0.297 long, 0.111 wide. Macrosetae: leg I: femur r1-p1, patella d1, tibia d2-r1-p1; leg II: femur r1, patella d1, tibia d2-r1; leg III: patella d1, tibia d1; leg IV: patella d1, tibia d1. Abdomen oval, wider than long (AL/AW 0.92). Abdomen covered with long and thin setae.

Coloration and markings (Fig. 6). Carapace, mouthparts, sternum, and legs dark yellowish brown (turning to yellowish brown in ethanol). Eyes on dark bases. Legs lacking annulation. Abdomen pale yellowish grey, dorsum of abdomen with two pairs of sigilla.

Palp (Figs 7, 9). Palpal patella with a strong dorsal macroseta. Paracymbium hook-like with a sharp tip. Cymbial lamella robust. Dorsal cymbial apophysis oblong, plate-like with blunt tip, extending anterior-retrolaterally. Tegulum large, bulbous, and occupying a large part of the palpal organ. Embolic division is a complex of long bristle-like apophyses, entirely covered with translucent conductor, and none of the embolic apophyses are exposed. Embolus short, blunt, and covered with a membrane. Three embolic apophyses conspicuous, EA 1 thickest, protruding middle of embolic division, S-shaped and sharper distally; EA 2 longest among them, bristle-like, basal part swelled, forming a loop at the ventro-prolateral side, distal part along with EA 1; EA 3 thinnest, protruding from prolateral side of embolic division. Median apophysis with a deep groove dividing it into two parts, distal translucent, weakly sclerotized and sharper ventrally, basal triangular, strongly sclerotized with narrower dorsally and spatula-like at ventral tip, MAL 1.07, MAW 0.66.

**Female** (paratype, NSMT-Ar 21723). Measurements. Body 2.37 long. Carapace 1.05 long, 1.06 wide, 0.65 high. Eye size and interdistances: AME 0.10, ALE 0.10, PME 0.12, PLE 0.09, AME-AME 0.02, AME-ALE 0.04, PME-PME 0.03, PLE-PLE 0.08. Leg length: leg I: 1.43 + 0.49 + 1.01 + 0.87 + 0.48 = 4.28; leg II: 1.22 + 0.45 + 0.85 + 0.70 + 0.40 = 3.62; leg III: 0.77 + 0.40 + 0.52 + 0.56 + 0.37 = 2.62; leg IV: 0.90 + 0.36 + 0.71 + 0.59 + 0.38 = 2.94. Abdomen 1.58 long, 1.45 wide, 1.49 high.

Carapace oval, as long as wide (CaL/CaW 0.99). Chelicerae with five teeth on promargin with the largest one positioned close to fang base, no teeth on posterior margin (Fig. 6H). Anterior eye row recurved, posterior eye row straight. Macrosetae: leg I: femur p1, patella d1, tibia d2-r1-p1; leg II: patella d1, tibia d2-r1; leg III: patella d1, tibia d1; leg IV: patella d1, tibia d1. Abdomen as in male (AL/AW 1.09).

Coloration and markings (Fig. 6). As in male.

Genitalia (Figs 8, 10). Epigyne a wide plate with an epigynal scape protruding from the posterior margin, epigynal scape spoon-like, and convex ventrally. Anterior margin of epigynal plate with a pair of dark-colored, sclerotized extensions protruding anteriorly from anterolateral side. Vulva. Spermatheca elliptical, longer laterally juxtaposed at the tip. Copulatory bursae developed. Course of copulatory ducts complicated: originating from copulatory bursae at ventral side, touching each other along the mesial line of the vulva, running posterior-dorsally under spermathecae, bend at a right angle toward laterally, curving anterior-dorsally at lateral side of vulva, forming a coil at lateral side of spermathecae, and then connecting to spermathecae. Fertilization ducts running under copulatory ducts and tip dorsally.

##### Variations.

There is a variation in the color of the abdomen: some individuals with dark grey abdomen, while others with pale yellowish grey abdomen. Course of embolic apophyses also varies among individuals: EA 2 tightly coiled with distal part along with EA 1 and EA 3 running below EA 1 in some individuals including the type specimens, while EA 2 loosely coiled with distal part apart from EA 1 and EA 2 running above EA 1.

##### Remarks.

Males and females are considered the same species because no other candidates were sympatric.

##### Distribution.

**Figure 11.**
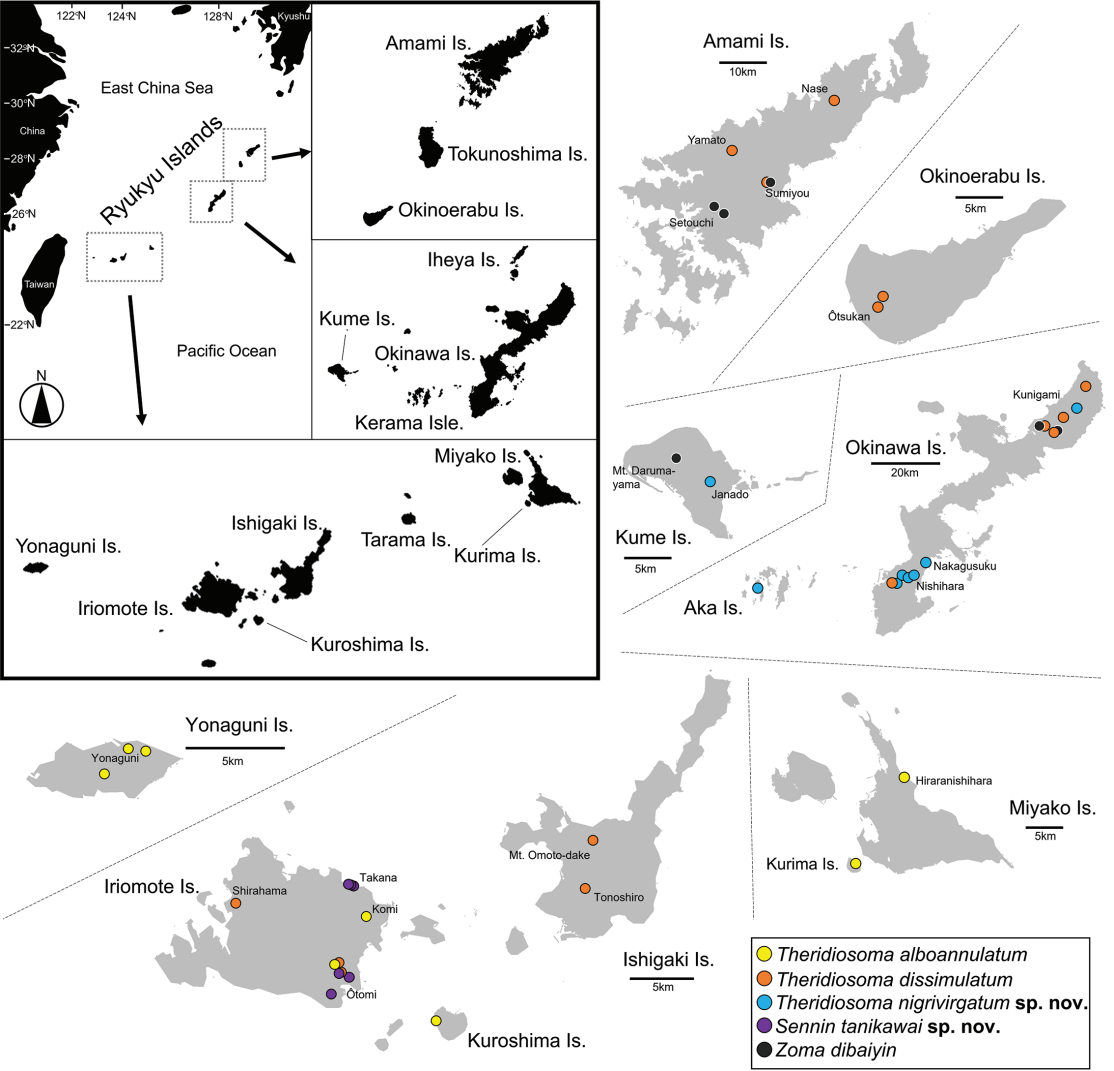
Distribution of theridiosomatid species in the Ryukyu Islands, Japan.

Japan (Iriomote Island; Fig. 11).

##### Habitat.

**Figure 12.**
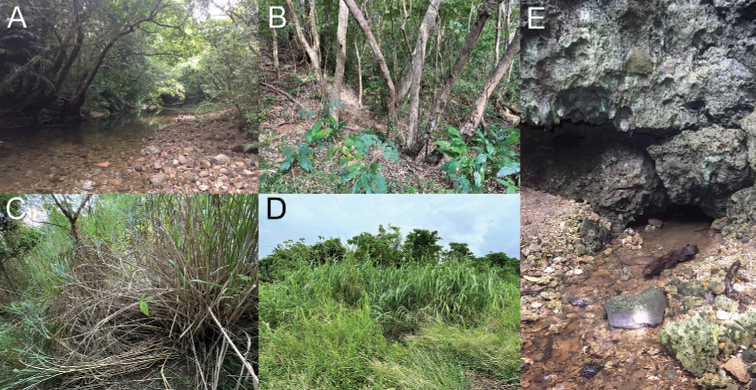
Habitats of theridiosomatid species in Ryukyu Islands **A** streamside in dim forest at Iriomote Island **B** forest floor of secondary forest at Kume Island **C** grassland at Kume Island **D** grassland at Yonaguni Island **E** crevices on limestone rocky wall at Iriomote Island.

The new species inhabits entrance or insides of limestone caves and crevices of limestone rocky walls (Fig. 12E). Spiders are found in high density at the entrance and twilight zones of humid caves, while sparsely deep inside the dark zone. Its general morphology (pigmented body, eight developed eyes, etc.) and habitat suggest that the species is troglophilic rather than obligate troglobite.

**Figure 13.**
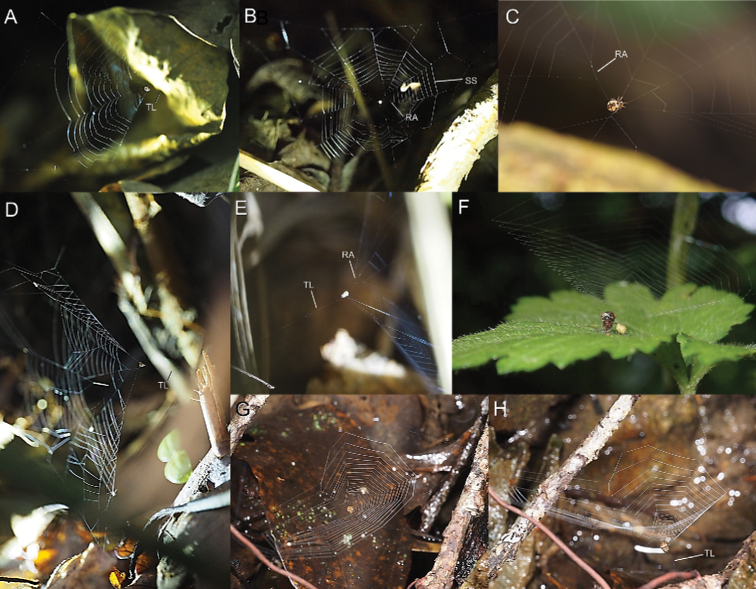
Web of *Theridiosoma* and *Zoma* spiders at Ryukyu Islands, Japan **A–C** web of *Theridiosomanigrivirgatum* sp. nov. **D–E** web of *Theridiosomaalboannulatum***F–H** web of *Zomadibaiyin*. Abbreviations: **RA** radial anastomosis **SS** sticky spirals **TL** tension line.

##### Web morphology.

**Figure 14.**
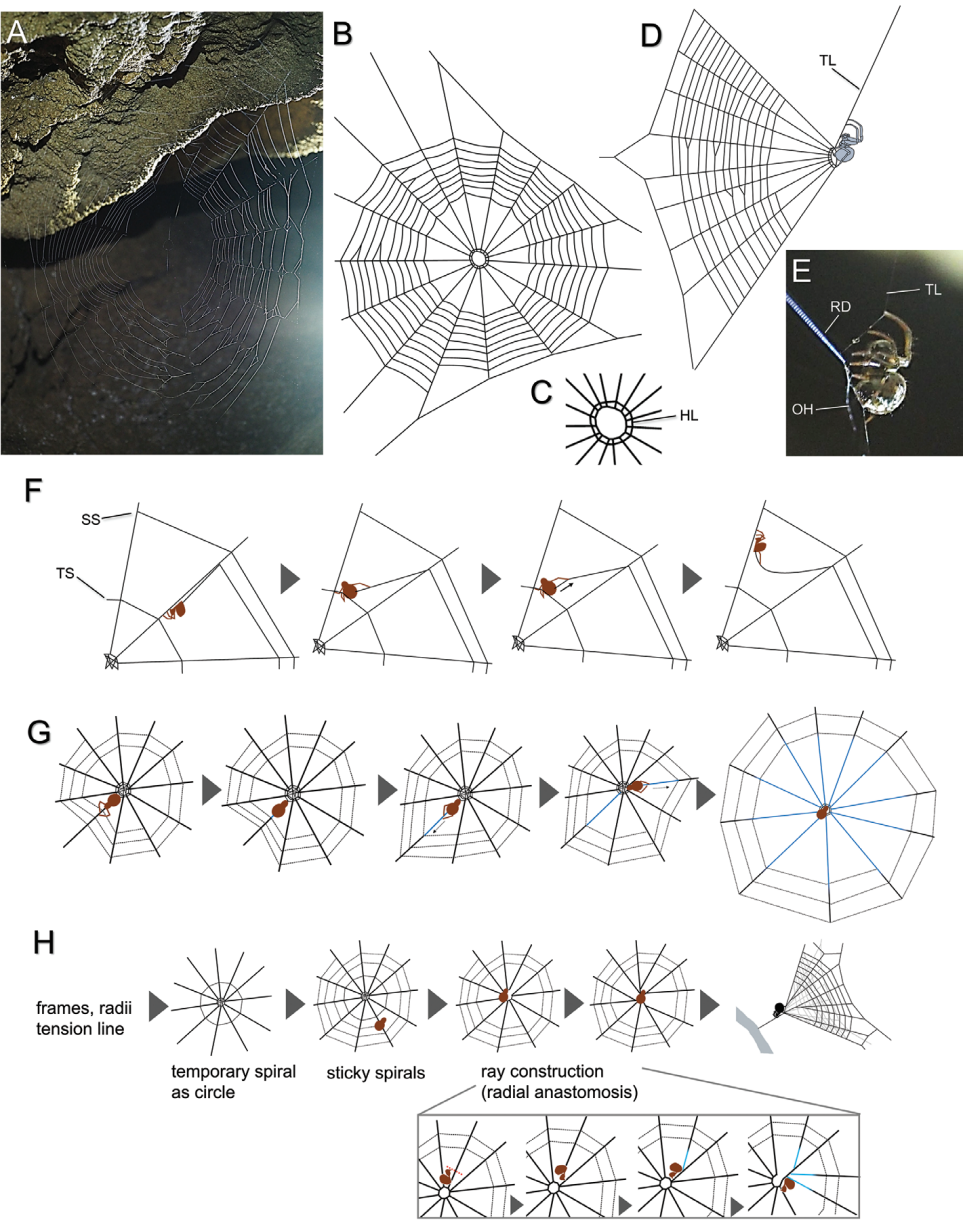
Web structure and building behavior in *Sennintanikawai* sp. nov. (**A–G**) and *Theridiosomaepeiroides* (**H**) **A** orb web, frontal view **B** orb web, illustrated **C** open hub, frontal view **D** orb web, lateral view **E** spider holding tension line with forelegs **F** process of weaving sticky spirals by *S.tanikawai* sp. nov. **G** radial elongation behavior in *S.tanikawai* sp. nov. **H** web building processes of *Theridiosomaepeiroides*. Blue lines indicate elongated portion of radii. Abbreviations: **HL** hub loops **OH** open hub **RD** radii **SS** sticky spiral **TL** tension line **TS** temporary spiral.

The newly reported species built a conventional orb web with an open hub and two hub loops (Fig. 14A–C). The web was almost vertical, and the tension line extended upward obliquely from the upper side of the hub to the surface of the rock (Fig. 14D). The angle of the trapline was approximately 60° to the horizontal plane. The spider sat upward and held a trapline by both forelegs and grasped radii by legs III, and put legs IV on the hub (Fig. 14E). The web turned conical shape (Fig. 14D), but it seemed to be less distorted than that of *Theridiosoma* spp. The mean web diameter was 11.6 × 10.1 (cm vertical × horizontal) (*n* = 9), number of radii: 17 ± 2.3 (SD), and number of sticky spirals: 14.5 ± 2.5 (SD) (*n* = 8). As a result of observation of 209 webs in June 2021, it was found that some individuals do not make tension lines. The percentage of webs with a tension line was as follows: female adult, 77% and juvenile, 33% in Yutsun-do Cave; female adult, 67% and juvenile, 45% in Ôtomi-Daiichi-Do Cave. Juveniles were more likely to build ordinary webs lacking tension lines than adults at both sites. When a web is disturbed by wind, the spider immediately escapes from the web running along the tension line (*n* = 22, see Suppl. material 1). After the escape, some spiders try to hide themselves into limestone rock crevices.

##### Web-building behavior.

**(*n* = 5)**. (1) Frames and radii were laid. (2) The spider returned to the hub and made a temporary spiral as a circle. (3) The spider pulled out a sticky line by using only the outer leg IV several times while touching the temporary spiral by the inner leg IV (in *T.epeiroides* Bösenberg & Strand, 1906, it draws out a sticky line using both legs IV alternately [Shinkai and Shinkai 1985]). (4) After drawing a sticky line, the spider walked to the frame along a radius holding it by the outer leg IV, shifted it inward, and then attached it to the radius (Fig. 14F; see Suppl. material 2). (5) The spider turned to the hub by drawing a new sticky line by the outer leg IV and moved the next radius along the temporary spiral (Fig. 14F). (6) The spider repeated sequences (3) to (5) and laid sticky spirals from outside to inside. (7) After finishing laying the sticky spirals, the temporary spiral was removed. (8) The spider moved near the hub and bit off the radius. (9) After biting off the radius, it changed the direction and attached its spinnerets to the radius and drew out a radius. The elongated radius was attached to the hub (Suppl. material 3). Thus, all radii were elongated (Fig. 14G). (10) The spider returned to the hub, laid two hub loops, and bit out its center. It ingested the ball of threads using both forelegs and digested it. (11) The spider held a tension line, and the web formed a cone. It took approximately one hour to complete the web.

##### Egg sac.

**Figure 15.**
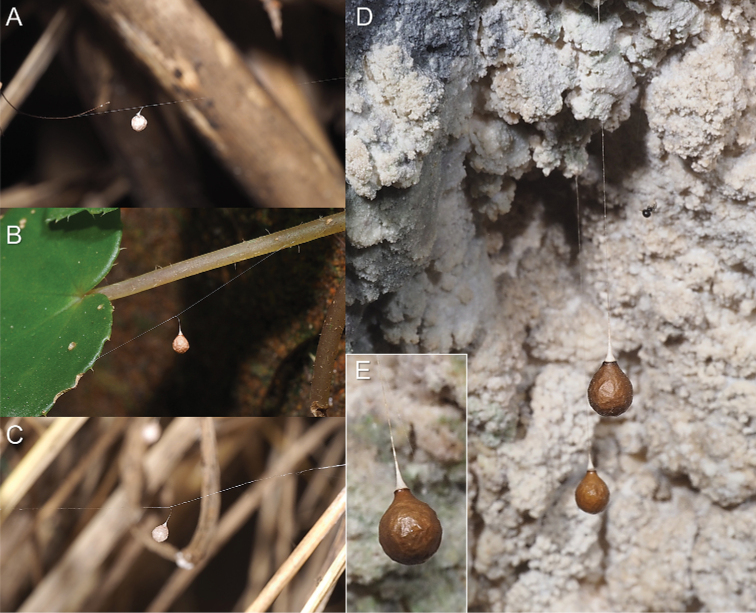
Egg sacs of theridiosomatid species **A** egg sac of *Theridiosomanigrivirgatum* sp. nov. (cap opened) **B** egg sac of *Theridiosomadissimulatum***C** egg sac of *Theridiosomaalboannulatum***D–E** egg sac of *Sennintanikawai* sp. nov.

Spherical and dark brown. The size was approximately 3 × 2 (mm, height × width), which was suspended with a long vertical line on the roof of a cave (Fig. 15D, E). This vertical line (pendant line) ranged from 2.5 to 5.3 cm, and the mean was 4.1 cm (*n* = 4). There was a single attachment point of the egg sac. The junction of the upper end of the egg sac and the lower end of the pendant line resembled a hatch, similar to a cap like structure in *Theridiosoma*. The lower end of the pendant line was thickened. The egg sac is cleaved at the joint. The egg sac of *K.upperyangtzica* resembles that of *S.tanikawai* sp. nov. but can be distinguished by the shape of ‘cap’ (thickened end of the pendant line): almost as long as wide in the former while clearly longer than wide in the latter (Chen 2010: fig. 29 vs. Fig. 15E).

#### ﻿ Sennin coddingtoni

Taxon classificationAnimaliaAraneaeTheridiosomatidae

(Zhu, Zhang & Chen, 2001) comb. nov.

BA8C12F5-782B-5544-9E05-6B99544F6D39

Wendilgarda coddingtoni Zhu, Zhang & Chen, 2001: 2, figs 1–7 (holotype female and paratypes from Liangxi Cave, Dongtang Village, Libo Country, Guizhou Prov., China; not examined).
Karstia coddingtoni : Chen 2010: 4, figs 15–28 (transferred from Wendilgarda).

##### Remarks.

See diagnosis section in *S.tanikawai* sp. nov.

##### Distribution.

China (Yunnan).

## ﻿Discussion

### ﻿Habitat and distribution

Although theridiosomatid species prefer dim and moist habitats, microhabitat preferences seem to differ among species. For example, among the Japanese *Theridiosoma* species, *T.epeiroides* prefers dim forests, while *T.fulvum* Suzuki, Serita & Hiramatsu, 2020 and *T.paludicola* Suzuki, Serita & Hiramatsu, 2020 mainly inhabit open and semi-aquatic environments such as wetlands, riverbeds, and pondside (Suzuki et al. 2020). In the Ryukyu Islands, both *T.dissimulatum* and *Z.dibaiyin* are predominantly collected from dim forests, whereas *T.nigrivirgatum* sp. nov. and *T.alboannulatum* were frequently found in open habitats such as grasslands (Fig. 12C, D), where the former two species are rarely found. Our survey revealed that *T.nigrivirgatum* sp. nov. is distributed on the Okinawa Islands, while *T.alboannulatum* is found on Miyako and Yaeyama Islands (Fig. 11), indicating that the distributional boundary of the two species can be found along the Tokara gap.

*Sennintanikawai* sp. nov. showed habitat preferences for limestone caves. Although troglophilic theridiosomatid species have never been reported in other regions of Japan, the congener *S.coddingtoni* comb. nov. is also known to inhabit the insides of limestone caves (Zhu et al. 2001; Chen 2010). Troglophilic species are more common in the neotropical and Chinese genera, for example, *Baalzebub* in China and Central America, *Alaria*, *Cuacuba*, *Karstia*, and *Sinoalaria* (Coddington 1986a; Chen 2010; Zhao and Li 2012, 2014; Prete et al. 2018; Prete and Brescovit 2020). Congeners of *Sennin* gen. nov. are expected to be found in the region between southern China and Iriomote Island, especially Taiwan.

### ﻿Web architecture and construction behavior of *S.tanikawai* sp. nov.

*Sennintanikawai* sp. nov. built a conventional orb with an open hub, resembling that of *Meta* (Araneae: Tetragnathidae). However, modification of the hub after the construction of sticky spirals, temporary spirals as circle, and elongation of radii clearly differentiate the new species from ordinary orb weavers (Tetragnathidae and Araneidae). Elongation of radii after spinning spirals is observed among tiny Araneoids of the families Anapidae, Symphytognathidae, and Mysmenidae (Shinkai and Shinkai 1985; Coddington 1986a,b; Eberhard 1987; Shinkai and Hiramatsu 2000), and is also seen in *Theridiosomaepeiroides* during the construction of radial anastomosis (as ‘ray’, Fig. 14H; also see Shinkai and Shinkai 1985). *Sennintanikawai* sp. nov. elongates all the radii without anastomosing, and it finally bites the hub as a hole and adds two hub loops (Fig. 14G). A series of radial elongation and hub construction behaviors has never been described in other theridiosomatids. The use of legs during the spinning of sticky spirals also differs between *S.tanikawai* sp. nov. and *T.epeiroides*. *Theridiosomaepeiroides* reels a sticky line out using both forth legs alternately, but *S.tanikawai* sp. nov. pulls out it by only outer leg IV while touching a temporary spiral as circle by inner leg IV (Fig. 14F). The significance of this difference in behavior of both species in web building is uncertain because details of the behavior are largely unknown in other theridiosomatid spiders. *Sennintanikawai* sp. nov. holds the radius away from its body with one leg IV after attaching a sticky line (Fig. 14F, also see Suppl. material 1). This behavior has also been reported in theridiosomatids (e.g., *Theridiosoma*, *Epeirotypus*, *Ogulnius*), and some anapids (*Anapis*, *Anapisona*) (Eberhard 1981). The function of this behavior is probably to avoid adhering sticky lines to the radius (Eberhard 1981).

Coddington (1986a) revised Theridiosomatidae mainly from neotropical and neosubtropical regions and discussed their natural histories, especially web morphology and web-building behaviors. The webs of *Epeirotypus* and *Naatlo* (Epeirotypinae) are typical orbs with tension lines, lack radial anastomosis, and hubs with two or more persistent hub loops (Table 1; also see Coddington, 1986a: figs 67, 69; Coddington 1986b: fig. 12.7), resembling the web of *S.tanikawai* sp. nov. Unlike *S.tanikawai* sp. nov., *Epeirotypus* species lacks radial elongation (Coddington 1986a). The web of *Baalzebub* also resembles that of *S.tanikawai* sp. nov. in appearance, but the former has a single hub loop without a tension line (Coddington 1986a: figs 165, 167), while the latter has two hub loops with a tension line. *Baalzebub* species adds a single hub loop after spinning sticky spirals, but the process of web building is unknown in detail. In appearance, the web of *S.tanikawai* sp. nov. is closer to those of *Epeirotypus* and *Naatlo* than that of *Baalzebub*. Based on the morphology of genitalia, *S.tanikawai* sp. nov. is closely related to *Baalzebub*. The subfamily Theridiosomatinae, to which *Baalzebub* belongs, is not closely related to Epeirotypinae in the cladistic analysis (Coddington 1986a). Therefore, the similarity in webs of two subfamilies might be the result of convergence. The multiple hub loops may contribute to the reinforcement of the central region of the web to defuse the tension by the ‘slingshot’ behavior. There are several differences in the webs and related behaviors of *S.tanikawai* sp. nov. and Epeirotypinae: the upward running of the tension line (downward in Epeirotypinae), upward posture of the spider on the hub (dorsal side up position in Epeirotypinae), quick escape behavior along the tension line (not observed in Epeirotypinae), and a ‘halfway’ slingshot posture (the web is more strongly distorted in Epeirotypinae). These characteristics suggest that the principal function of the tension line in this species may be to escape from any predator, and the function of prey-capture may be secondary. The main predator of *S.tanikawai* sp. nov. is unknown. Bat (Chiroptera) is one of the major predators inhabiting caves, but they are less likely to forage for this species, as all cave-dwelling bats reported on Iriomote Island forage insects outside caves (Okinawa Prefectural Board of Education 1985). *Plato* is confirmed to have no tension line (Coddington 1986a; Eberhard 2020), while the presence or absence of tension lines has not been examined in most cave-dwelling theridiosomatids: *Cuacuba*, *Karstia*, and *Sinoalaria* (Chen 2010; Zhao and Li 2014; Prete et al. 2018). If these cave-living theridiosomatids also lack a tension line, it would be interesting to know whether or how they perform escape behavior.

**Table 1.** Comparison of habitat and web morphology of theridiosomatid genera of which web morphology were described in published papers. Data source: ^a^Coddington (1986a), ^b^Eberhard (1986), ^c^Eberhard (2020), ^d^Chen (2010), ^e^Shinkai and Shinkai (1985), ^f^Hiramatsu (2021). + = present; -= absent, ? = unknown.

Subfamily	Genus	Habitat	Orb web	Web shape	Web angle	Tensi-on line	Angle of tension line	Radial anasto-mosis	Open hub	Hub loops
Platoninae	*Chthonos* ^a^	leaf litter^1^	-	no web	-	-	-	-	-	-
	*Plato* ^a^	caves, dark places	+	loose orb web	vertical or diagonal	-	?	+	-	-
Epeiroty-pinae	*Epeirotypus* ^a, b, c^	shrubs, shaded wet forest	+	concave orb web	vertical or diagonal	+	almost horizontal or downward	-	+	2–5
	*Naatlo* ^a^	humid shaded forest	+	concave orb web	vertical or diagonal	+	?	-	+	2
Ogulniinae	*Ogulnius* ^a, b^	wet, shaded forest	-	sparse network	-	-	-	-	-	-
Theridio-somatinae	*Baalzebub* ^a^	interior of hollow logs, under fallen tree, caves	+	ordinary orb web	vertical or diagonal	-	-	-	+	1
	*Epilineutes* ^a^	over stream water	+	conventional orb web	vertical or diagonal	+ (rare)	?	+	-	-
	*Karstia* ^d^	limestone caves	+	?	vertical?	?	?	?	?	?
	*Sennin* gen. nov.	limestone caves	+	conventional orb web	vertical or diagonal	+	almost upward, sometimes horizontal	-	+	2
	*Theridiosoma* ^a, c, e^	wet, shaded forest, etc.	+	concave orb	vertical or diagonal	+	almost horizontal or downward	+	-	-
	*Wendilgarda* ^c^	over stream water or ponds	-	Naruko web	-	-	-	-	-	-
	*Zoma* ^f^	wet, shaded forest	+	concave orb	horizontal	+	vertical to the ground	+	-	-

As the tension line is sporadic throughout theridiosomatids (Coddington 1986a), the origin and function of the tension line can vary (for example, Eberhard 2020: fig. 9.4.(d)(e)). Further morphological and molecular analyses of theridiosomatids, including *Sennin* gen. nov. species, are expected to elucidate the evolutionary process of the tension line.

## Supplementary Material

XML Treatment for Theridiosoma

XML Treatment for Theridiosoma nigrivirgatum

XML Treatment for Theridiosoma dissimulatum

XML Treatment for Theridiosoma alboannulatum

XML Treatment for Zoma

XML Treatment for Zoma dibaiyin

XML Treatment for Sennin

XML Treatment for Sennin tanikawai

XML Treatment for Sennin coddingtoni

## Supplementary materials

Supplementary material 1 Video S1 Data type Video (mp4. file). Explanation note *Sennintanikawai* sp. nov. escaping from the web via a tension line. https://binary.pensoft.net/file/708582This dataset is made available under the Open Database License (http://opendatacommons.org/licenses/odbl/1.0/). The Open Database License (ODbL) is a license agreement intended to allow users to freely share, modify, and use this Dataset while maintaining this same freedom for others, provided that the original source and author(s) are credited.Yuya Suzuki, Takehisa Hiramatsu, Haruki Tatsuta

Supplementary material 2 Video S2 Data type Video (mp4. file). Explanation note SS weaving behavior in *Sennintanikawai* sp. nov. https://binary.pensoft.net/file/708583This dataset is made available under the Open Database License (http://opendatacommons.org/licenses/odbl/1.0/). The Open Database License (ODbL) is a license agreement intended to allow users to freely share, modify, and use this Dataset while maintaining this same freedom for others, provided that the original source and author(s) are credited.Yuya Suzuki, Takehisa Hiramatsu, Haruki Tatsuta

Supplementary material 3 Video S3 Data type Video (mp4. file). Explanation note Radial elongation behavior in *Sennintanikawai* sp. nov. https://binary.pensoft.net/file/708584This dataset is made available under the Open Database License (http://opendatacommons.org/licenses/odbl/1.0/). The Open Database License (ODbL) is a license agreement intended to allow users to freely share, modify, and use this Dataset while maintaining this same freedom for others, provided that the original source and author(s) are credited.Yuya Suzuki, Takehisa Hiramatsu, Haruki Tatsuta
